# Glycofullerene–corrole hybrids: a new class of multifunctional nanomaterials with potential in targeted photodynamic therapy

**DOI:** 10.1039/d5sc06977g

**Published:** 2025-12-08

**Authors:** Jennifer Patino-Alonso, Carla I. M. Santos, Adriana F. Cruz, Sandra Pinto, Justo Cabrera-González, M. Amparo F. Faustino, M. Graça P. M. S. Neves, Ermelinda M. S. Maçôas, Nazario Martín, Beatriz M. Illescas

**Affiliations:** a Departamento de Química Orgánica, Facultad de Química, Universidad Complutense E-28040 Madrid Spain beti@ucm.es nazmar@ucm.es; b Centro de Química Estrutural, Institute of Molecular Sciences, Departamento de Engenharia Química, Instituto Superior Técnico, Universidade de Lisboa 1049-001 Lisboa Portugal carla.santos@tecnico.ulisboa.pt; c LAQV-REQUIMTE and Department of Chemistry, University of Aveiro, Campus Universitário de Santiago 3810-193 Aveiro Portugal; d iBB-Institute for Bioengineering and Biosciences, Instituto Superior Técnico Av. Rovisco Pais 1049-001 Lisboa Portugal; e Departamento de Química en Ciencias Farmacéuticas, Facultad de Farmacia, Universidad Complutense E-28040 Madrid Spain; f IMDEA-Nanoscience, Campus de Cantoblanco C/ Faraday 9 28049 Madrid Spain

## Abstract

Corrole photosensitizers for photodynamic therapy (PDT) have garnered significant attention due to their promising potential in cancer treatment. Advances in understanding their photophysical and photochemical properties have facilitated the development of more efficient and targeted PDT strategies. This study reports the synthesis and characterization of a series of alkyne-substituted gallium(iii) corrole complexes, as well as their conjugation to azide-functionalized glycofullerenes *via* copper-catalyzed azide–alkyne cycloaddition (CuAAC) reactions. The resulting glycofullerene–corrole conjugates were obtained and fully characterized using standard spectroscopic techniques. Their photodynamic efficacy was evaluated *in vitro* using HeLa cells. Among the series, the mono- and tris-alkyne-substituted corroles, as well as the monofunctionalized glycofullerene conjugate, exhibited the most potent PDT effects in cells, achieving IC_50_ values below 1.0 µM under blue irradiation at 420 nm with a total light dose as low as 5 J cm^−2^. These findings highlight the potential of gallium(iii) corrole-based nanostructures as water-soluble and efficient photosensitizers for PDT applications.

## Abstract 2

Corrole photosensitizers for photodynamic therapy (PDT) have garnered significant attention due to their promising potential in cancer treatment.

## Graphical abstract

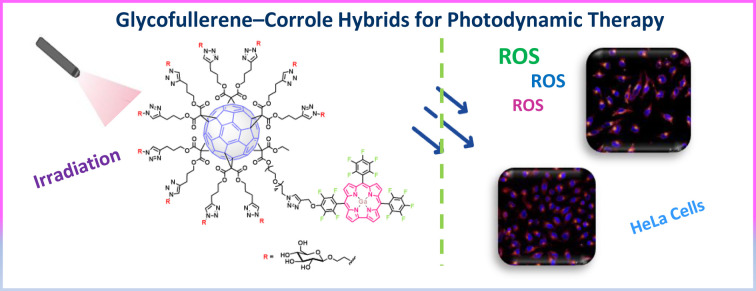

## 1. Introduction

Cancer's high mortality rate makes it one of the most challenging and fatal diseases globally. According to the latest estimates from the International Agency for Research on Cancer GLOBOCAN 2022 database, approximately 20 million new cases of cancer and close to 10 million cancer deaths were estimated worldwide in 2022.^1^ As a result, there is an urgent need for novel diagnostic techniques and effective treatments to address this complex disease. In contrast to traditional therapies such as surgery, radiotherapy, and chemotherapy, photodynamic therapy (PDT) has garnered significant attention due to its ability to achieve precise spatiotemporal control, low invasiveness, and minimal side effects. PDT is based on the topical or systemic application of a dye known as a photosensitizer (PS), that, when locally activated by light of an appropriate wavelength in the presence of dioxygen (^3^O_2_), produces reactive oxygen species (ROS) that trigger a sequence of chemical and photobiological reactions, capable of inducing selective cell death.^2–4^ Ideally, the PS should exhibit absorption within the therapeutic window (620–850 nm), possess high photostability, and present selective uptake and accumulation in cancer cells with minimal dark toxicity.^5^ Corroles, porphyrinoids characterized by the absence of one methine (=CH–) bridge compared to porphyrins, have emerged as promising PS for PDT.^6–12^ These contracted tetrapyrrolic macrocycles show unique properties, such as low symmetry, high molar extinction coefficients, fluorescence emission within the therapeutic window, and ability to generate singlet oxygen (^1^O_2_), which make them attractive therapeutic agents for PDT.^13–15^ Among them, gallium(iii) corroles have demonstrated effectiveness in tumor detection and therapy, due to their relatively high fluorescence quantum yield and efficient ROS generation. The insertion of gallium(iii) into the corrole core enhances the rate of intersystem crossing (ISC) and promotes the efficient generation of ^1^O_2_ (ref. 10). In early studies, developed by Gross *et al.*, it was demonstrated that sulfonated gallium(iii) corroles functionalized with protein carriers (HerGa) are efficient in PDT and fluorescence imaging. The resulting macrocycles exhibited good cytotoxicity against several cancer cell lines and generated superoxide, which led to disruption of the cytoskeleton and mitochondria.^16,17^ In 2018, Zeng *et al.* reported the synthesis of the gallium(iii) complex of 5,10,15-tris(ethoxycarbonyl)corrole and its coupling with a monoclonal antibody (mAb) targeting CT83. The obtained conjugate displayed efficient photodynamic activity for the selective treatment of CT83-expressing cancer.^18^ Recently, the group of Liu *et al.* demonstrated that gallium(iii) corroles with various substituents such as hydroxyl groups, coumarins and azides, could induce cancer cell apoptosis, due to the increased intracellular ROS and the disruption of the mitochondrial membrane potential.^8,19,20^ Despite the previously mentioned advances in the development of corroles as PS, some obstacles to their progress are still present, such as their low water solubility, as well as their lack of selective delivery and specificity to cancer cells. Conjugation of corroles to nanomaterials has been explored as a strategy to address these challenges.^21–23^ Soy *et al.* reported the synthesis and characterization of phosphorus(v) and gallium(iii) complexes of an A_3_ triarylcorrole with 4-methylthiophenyl *meso*-groups, as well as the formation of new conjugates resulting from their coupling with gold nanoparticles (AuNPs). The photodynamic activities of the resulting hybrid nanomaterials were assessed in MCF-7 breast cancer cells. Upon conjugation to AuNPs, the fluorescence quantum yields of the phosphorus(v) and gallium(iii) corroles decreased, while the ^1^O_2_ quantum yields increased due to an external heavy atom effect. In addition, the P(v) complex and their AuNP conjugates displayed more favorable PDT activity than gallium(iii) derivatives.^10^ Conjugation of corroles with biocompatible carbon nanostructures, as graphene quantum dots or nanodiamonds, have also been studied. In these conjugates, the photoluminescence properties are additive with respect to the absorption and emission characteristics of the constituent moieties. The cellular uptake of the hybrids is confirmed and therefore they hold promise for different biological applications, as fluorescent labels, photodynamic and photothermal therapy agents or cancer theragnostic platforms for bioimaging.^24,25^

In the present study, the synthesis of alkyne-substituted corroles and their subsequent conjugation with glycofullerene derivatives through copper-catalyzed azide–alkyne cycloaddition (CuAAC) reaction is explored. While numerous glyco-conjugates of porphyrinoids with carbohydrates have been studied,^26,27^ examples involving glycocorroles remain scarce.^28,29^ The conjugation of corroles with glycofullerenes is anticipated to enhance their biocompatibility, water solubility and reduce cytotoxicity, making the resulting materials suitable for biomedical applications.^30–32^ Carbohydrates are not only expected to increase water solubility, as they also play important biological roles. In our previous work, porphyrins functionalized with four glycofullerene moieties showed an efficient inhibition of SARS-CoV-2 *trans*-infection process by competitively interacting with DC-SIGN (dendritic cell-specific intercellular adhesion molecule-3 grabbing non-integrin).^33^ Glycofullerenes themselves have demonstrated significant anti-proliferative effects against various cancer cell lines.^34,35^ These sugar-functionalized fullerene derivatives exhibit enhanced selectivity toward malignant cells, potentially improving therapeutic efficacy while minimizing off-target effects. In some cases, they also enable targeted delivery of the fullerene core to tumor tissues. This work aims to develop a synthetic protocol of a series of novel glycofullerene–corrole conjugates and determine their photochemical and photophysical properties, as well as their PDT activity toward HeLa tumor cells.

## 2. Results and discussion

**Scheme 1:**
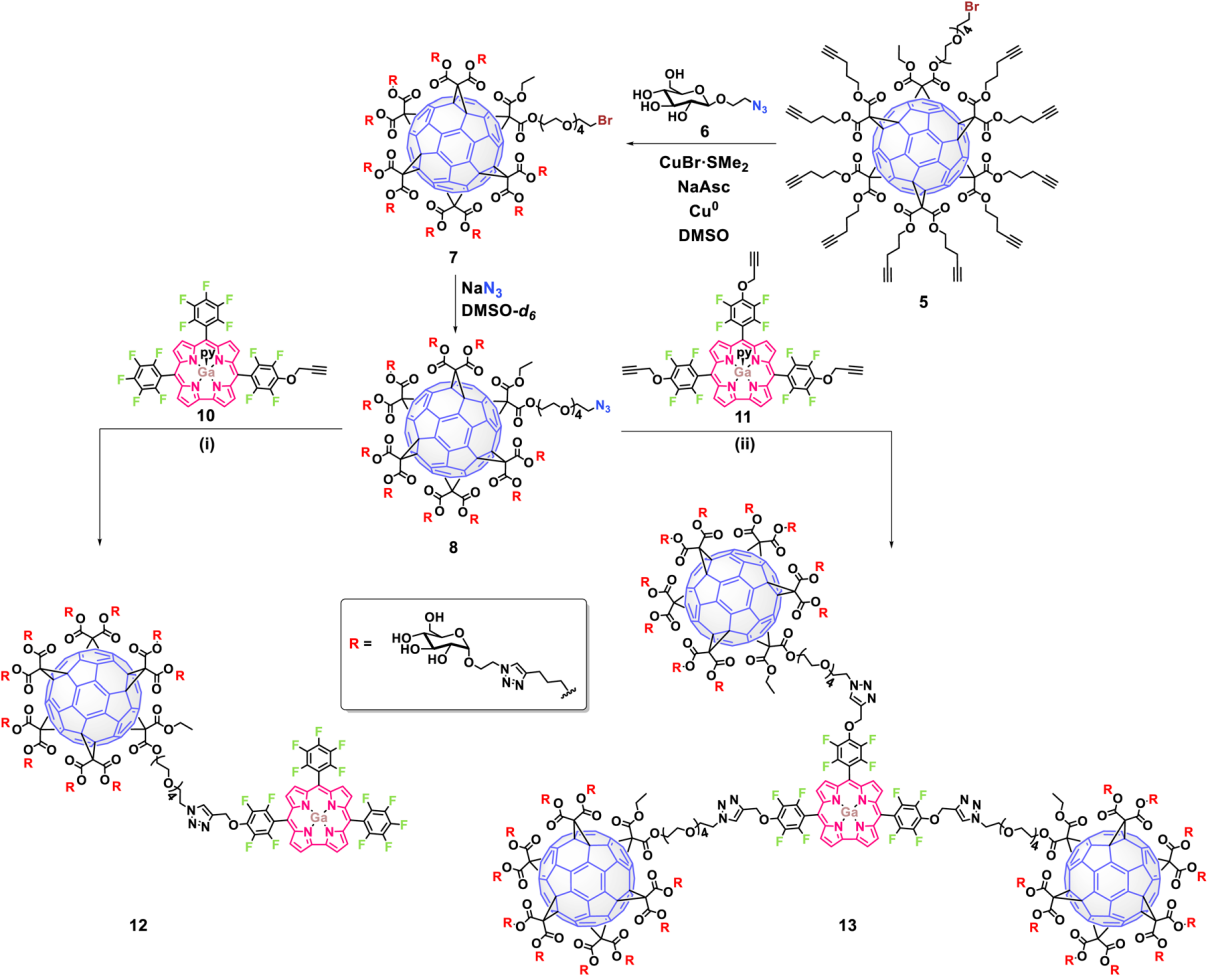
Syntheses of glycofullerene–corrole conjugates 12 and 13 using Click chemistry. Reagents and conditions: (i) CuBr·S(CH_3_)_2_, sodium ascorbate, Cu^0^, DMSO, 48 h (74%); (ii) CuBr·S(CH_3_)_2_, sodium ascorbate, Cu^0^, DMSO, 72 h (81%). In the case of compound 10, just the major isomer has been represented.

The synthetic strategy to obtain the new glycofullerene–corrole conjugates 12 and 13 (Scheme 1) was based on a click-chemistry methodology, following our previously reported synthetic protocol.^33^ The approach aimed to create an asymmetric [5 : 1] fullerene hexakis-adduct, designed for orthogonal functionalization, which allowed for the initial attachment to carbohydrates followed by subsequent linkage to corroles (Scheme S1). The synthesis began with the esterification of monobromo-pentaethylene glycol (PEG 1) (Fig. S1 and S2) with ethyl malonyl chloride, followed by stepwise Bingel–Hirsch cyclopropanation reactions of the resulting malonate 2 (Fig. S3 and S4) and di(pent-4-yn-1-yl) malonate 4 with fullerene C_60_, affording the hexakis-adduct 5 in good yield (71%) (Fig. S5–S7). Glucose-azide derivative 6 was then attached to complementary alkynes in 5 through a CuAAC reaction, followed by the elimination of copper to prevent interference in biological studies. Finally, the nucleophilic substitution of terminal bromine in the resulting intermediate 7 (Fig. S8–S10) with sodium azide under microwave (MW) irradiation afforded the building block 8 in high yield (93%) (Fig. S8–S13).

The synthesis of alkyne-substituted corroles 10 and 11 involved the controlled nucleophilic substitution of the *para*-fluorine atoms of the gallium(iii) complex of 5,10,15-tris(pentafluorophenyl)corrole (9) with propargyl alcohol (Scheme S2). By adjusting the equivalents of propargyl alcohol and the reaction conditions, the monosubstituted (10) or trisubstituted (11) products were selectively obtained. Thus, using only a slight excess of propargyl alcohol at 100 °C in DMSO for 3 h and K_2_CO_3_ as the base, the monosubstituted corroles 10 were preferentially obtained. Differently from our previous reported glycol substituted corroles,^24^ the alkyne functionalized corroles 10a and 10b were obtained as an inseparable mixture of isomers by flash chromatography or preparative TLC. However, both regioisomers could be distinguished by ^1^H NMR and ^19^F NMR spectroscopy (Fig. S14–S17) revealing that 10a and 10b are present in a 2 : 1 ratio, respectively.

To obtain the triply functionalized corrole 11, a slight excess of propargyl alcohol, previously treated with NaH, was added to the fluorinated corrole 9 and the reaction mixture was kept under reflux in THF for 1 h. After work-up and chromatographic purification, the desired derivative was isolated in 56% yield (Fig. S18–21).

The conjugation of alkyne-substituted Ga(iii) corrole complexes to the azide-functionalized glycofullerene 8 was performed *via* CuAAC reactions (Scheme 1). Under these conditions, the glycofullerene–corrole conjugate 12 was obtained using the inseparable regioisomers 10a and 10b, while conjugate 13 was obtained in the presence of corrole 11. The resulting conjugates 12 and 13, which contain 10 and 30 glucose units, respectively, were isolated with yields exceeding 70% (Fig. S22–S29).

The characterization of these new nanostructures was carried out by standard spectroscopic techniques. Fullerene hexakis-adducts can be easily characterized using ^13^C NMR due to the high symmetry of the molecule with the appearance of only two signals corresponding to the sp^2^ carbons of the C_60_ core at ∼140 and 145 ppm (Fig. S6). In the case of derivative 7, its correct functionalization with glucose is unequivocally confirmed through ^13^C NMR by the appearance of the signal at ∼102 ppm assigned to the anomeric carbon of the glucose moiety and two new signals at ∼123 and 146 ppm corresponding to the new triazole rings formed during the click reaction (Fig. S9).

The substitution of bromine by azide to obtain compound 8 can also be tracked down by ^13^C NMR under the disappearance of the corresponding *C*–Br signal at ∼32 ppm and the appearance of the new *C*–N_3_ resonance at ∼50 ppm (Fig. S12). Additionally, using infrared spectroscopy, the appearance of the azido band at 2108 cm^−1^ can be detected (Fig. S13).

**Fig. 1:**
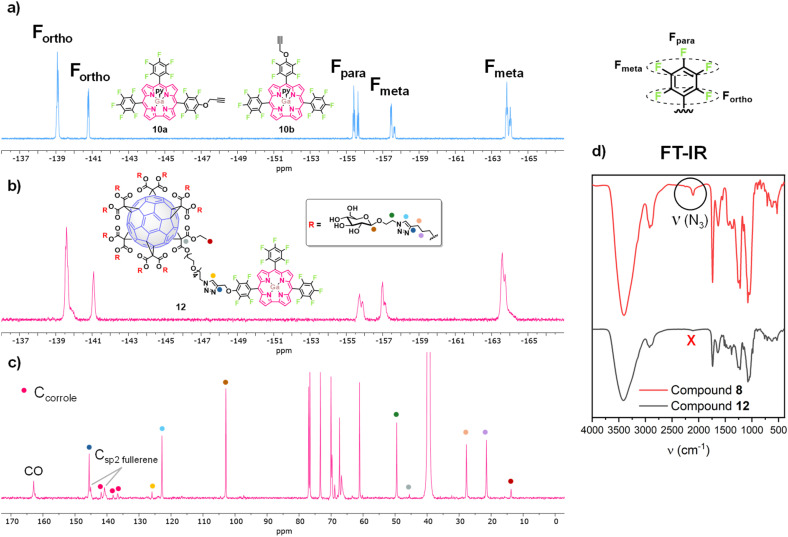
(a) ^19^F NMR spectrum of 10 (CD_2_Cl_2_, 471 MHz). (b) ^19^F NMR spectrum of 12 (CD_2_Cl_2_, 471 MHz). (c) Partial view of the ^13^C NMR spectrum of 12 (DMSO-*d*_6_, 176 MHz). (d) FT-IR of 8 and 12.

Regarding the characterization of the new glycofullerene–corrole conjugates 12 and 13, ^19^F NMR spectra show signals corresponding to fluorine atoms from the corrole moiety, indicating successful functionalization (Fig. 1, S24 and S28). Moreover, the ^1^H NMR spectra show new peaks in the aromatic region corresponding to the resonance of β-H of the corrole core and a broad singlet at ∼5.7 ppm corresponding to the C*H*_2_ of the propargyl linker (Fig. S22 and S26). Additionally, two signals corresponding to the two different triazole rings formed in the click reaction are also observed, one at *δ* ∼ 8.5 ppm integrating for 1H in compound 12 and 3H in compound 13, and another at *δ* ∼ 7.9 ppm integrating for 10H and 30H for compounds 12 and 13 respectively, as expected, considering the number of fullerene units in each hybrid. Once again, ^13^C NMR allows the complete characterization of the molecules. The disappearance of the *C*–N_3_ signal of the starting glycofullerene 8, accompanied by the emergence of two distinct signals corresponding to the resonance of each type of triazole ring in the structure (*δ* ∼ 146 ppm and 123 ppm for the triazole ring of the glycofullerene moiety and *δ* ∼ 146 ppm and 126 ppm for the triazole ring connecting the corrole with the glycofullerenes) as well as minor signals in the aromatic region, confirm both the presence and correct functionalization of the corroles (Fig. 1, S23 and S27). Despite comprehensive NMR characterization, the molecular ion peaks of compounds 12 and 13 could not be observed in their mass spectra, likely due to the extensive fragmentation typically associated with these compounds.^30^

DOSY (Diffusion-Ordered NMR Spectroscopy) NMR experiments (∼2 mg mL^−1^ in D_2_O, Fig. S30 and S31) and DLS (Dynamic Light Scattering) measurements (Fig. S32 and S33) were carried out to estimate the overall molecular size of conjugates 12 and 13. These techniques enable the calculation of the hydrodynamic radius (*R*_H_) of the molecules. Molecular weights (MW), diffusion coefficient (*D*) and hydrodynamic radius (*R*_H_) values are shown in Table 1. It can be observed that there is a correlation between the molecular weight and the diffusion coefficient, where an increase in the size of the molecule between 12 (*R*_H_ = 38.9 Å) and 13 (*R*_H_ = 42.1 Å) is observed as anticipated.

**Table 1:** Diffusion coefficients and hydrodynamic radii determined by DOSY

Compound	Molecular weight (MW)	Diffusion coefficient (*D*, m^2^ s^−1^)	Hydrodynamic radii (*R*_H_, Å)
12	5658.70	6.30 × 10^−11^	38.9
13	15 249.75	5.82 × 10^−11^	42.1

### 2.1 Photophysical studies

**Fig. 2:**
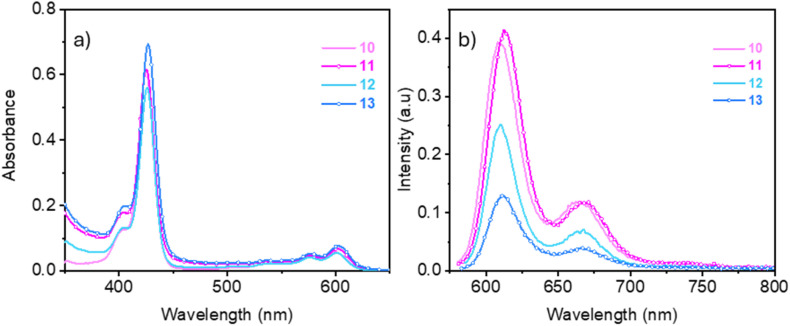
UV-Vis absorption and emission spectra of the corroles (10 and 11) and glycofullerene–corrole conjugates (12 and 13) in DMSO (5 × 10^−6^ M): (a) absorption spectra and (b) fluorescence emission spectra excited at 575 nm.

The photophysical characterization of the glycofullerene–corrole conjugates 12 and 13 was performed in water, DMF and DMSO at room temperature and the spectra of their precursors 10 and 11 were evaluated in DMSO and DMF. The absorption and emission spectra of all compounds in DMSO are shown in Fig. 2 and in the remaining solvents are depicted in Fig. S34 and S35. The recorded spectroscopic data are summarized in Table 2. No significant spectral differences were observed across the various solvents evaluated. All compounds exhibit the typical absorption spectrum of corrole, including a strong Soret band at ∼425 nm and several weak Q bands between 500–660 nm.^36^ The UV-Vis absorption spectra of the glycofullerene–corrole conjugates 12 and 13 appear dominated by the absorbance of the corrole unit with no major shifts observed upon conjugation to the glycofullerene. This observation suggests that there is no strong interaction between the two moieties in the ground state.

**Table 2:** Photophysical and photochemical properties of glycofullerene–corrole conjugates 12 and 13 and their precursors 10 and 11

Comp.	Solvent	Soret/nm (*ε*/10^3^ M^−1^ cm^−1^)	Q-Bands/nm (*ε*/10^3^ M^−1^ cm^−1^)	*λ* ^max^_em_/nm	Stokes shift (nm) (Q_2_-*λ*^max^_em_)	*ϕ*_F_ a (%)	*Φ*_Δ_ b (%)	*τ*_r_ (ns)
10	DMF	423 (228.7)	574 (17.7)	605	7	8.9	60	1.8
			598 (24.6)	660				
	DMSO	425 (219.9)	575 (17.7)	608	9	7.8	—	—
			599 (20.1)	665				
11	DMF	424 (231.5)	574 (17.6)	608	8	9.9	65	1.8
			600 (23.9)	663				
	DMSO	427 (231.7)	576 (18.7)	612	9	8.7	—	—
			603 (22.8)	666				
12	DMF	424 (212.6)	575 (15.6)	609	10	2.3	61	1.7
			599 (20.3)	661				
	DMSO	426 (207.2)	576 (16.6)	610	9	1.6	—	—
			601 (19.9)	668				
	H_2_O	423 (114.0)	574 (8.2)	606	11	—	—	—
			595 (12.2)	657				
13	DMF	425 (215.3)	576 (17.1)	611	9	1.2	30	0.7
			602 (25.4)	666				
	DMSO	427 (244.5)	577 (20.3)	612	8	1.4	—	—
			604 (26.5)	666				
	H_2_O	425 (222.1)	576 (22.9)	606	8	—	—	—
			598 (29.0)	660				

a Fluorescence quantum yields (*Φ*_F_) were measured by the reference method using a cresyl violet perchlorate solution in methanol (*Φ*_F_ = 0.54) as ref. 45. The quantum yields in water are not reported because a reduction by two orders of magnitude was observed.

b Singlet oxygen quantum yields (*Φ*_Δ_) were determined with TPP in DMF (*Φ*_Δ_ = 0.65) as ref. 46.

The starting corroles 10 and 11, along with the glycofullerene conjugates derivatives 12 and 13 show fluorescence emission values peaking at *ca* 610 nm (Fig. 2b and Table 2) characteristic of the corrole unit. The glycofullerene moieties in conjugates 12 and 13 do not contribute to the observed emission spectra. This observation aligns with the non-emissive nature of glycofullerene 8, confirming that the fluorescence properties of the conjugates are primarily attributed to the corrole component. As shown in Table 2, glycofullerene–corrole conjugates 12 and 13 and the starting alkyne corroles 10 and 11 have significant Stokes shift which make them suitable for application in bioimaging where self-quenching is minimized and the signal-to-noise ratio is also reduced.^37^

The fluorescence quantum yield in DMF of the alkyne substituted corroles 10 and 11 (*Φ*_F_ = 8.9 and 9.9%) are significantly higher than those of the corresponding glycofullerene–corrole conjugates 12 and 13 (*Φ*_F_ = 2.3 and 1.2% respectively) (Table 2). The radiative lifetime (*t*_r_) of the corrole 10 (1.8 ns) is only slightly affected by conjugation with glycofullerene in the conjugate 12 (1.7 ns) (Fig. S36). Conversely, radiative lifetime of corrole 11 (1.8 ns) decreases in glycofullerene–corrole conjugate 13 (0.7 ns), concomitantly with the reduction in the emission quantum yield. From the radiative lifetime and the fluorescence emission quantum yield, we can estimate the radiative relaxation rate (*k*_r_ = *Φ*_F_/*t*_r_) and the non-radiative relaxation rate (*k*_nr_ = (1/*t*_r_) − *k*_r_). The emission lifetimes are largely determined by the non-radiative relaxation rates (5 × 10^8^ to 14 × 10^8^ s^−1^) that are one order of magnitude higher than the radiative relaxation rate (1× 10^7^–5 × 10^7^ s^−1^). Conjugation of a single glycofullerene unit increased only slightly the non-radiative relaxation rate that changed from 5.2 × 10^8^ s^−1^ in 10 to 5.7 × 10^8^ s^−1^ in 12. Conversely, conjugating simultaneously three glycofullerene units to corrole 11 increases the non-radiative relaxation rate by a factor of three, from 5 × 10^8^ (11) to 14 × 10^8^ s^−1^ (13). Internal conversion followed by vibrational cooling and/or intersystem crossing followed by singlet oxygen (^1^O_2_) generation are two of the possible competing non-radiative relaxation pathways that could be enhanced in the hybrids.

The ^1^O_2_ quantum yield (*Φ*_Δ_) in DMF of 10, 11, 12, and 13 were measured as 60, 65, 61 and 30%, respectively. The singlet oxygen generation remains as efficient in conjugate 12, bearing a single glycofullerene unit, as in the corroles 10 and 11. Upon conjugation of three glycofullerene units in conjugate 13, the *Φ*_Δ_ value decreases by half relative to the alkyne functionalized corroles. The high local concentration of glycofullerenes around the corrole seems to contribute to reduce the singlet oxygen generation yield in conjugate 13. Fullerenes are well-known ROS generators under irradiation, but they are also recognized for their radical-scavenging properties in the ground state.^38–42^ Alternatively, the simultaneous conjugation of three glycofullerenes to the corrole could affect the external heavy atom effect promoted by gallium(iii). Gallium(iii) is a relatively heavy atom that enhances the rate of ISC to the triplet state from where ^1^O_2_ is generated.^43,44^ The decrease in the *Φ*_Δ_ value of conjugate 13 allows us to exclude singlet oxygen generation as one of the pathways contributing to the increased non-radiative relaxation rate in the hybrids. Alternatively, efficient internal conversion and vibrational cooling facilitated by the increased density of vibrational states arising from the glycofullerene units seems to be a more relevant non-radiative relaxation mechanism.

### 2.2 Biological assays

#### 2.2.1 Cytotoxicity assays

**Fig. 3:**
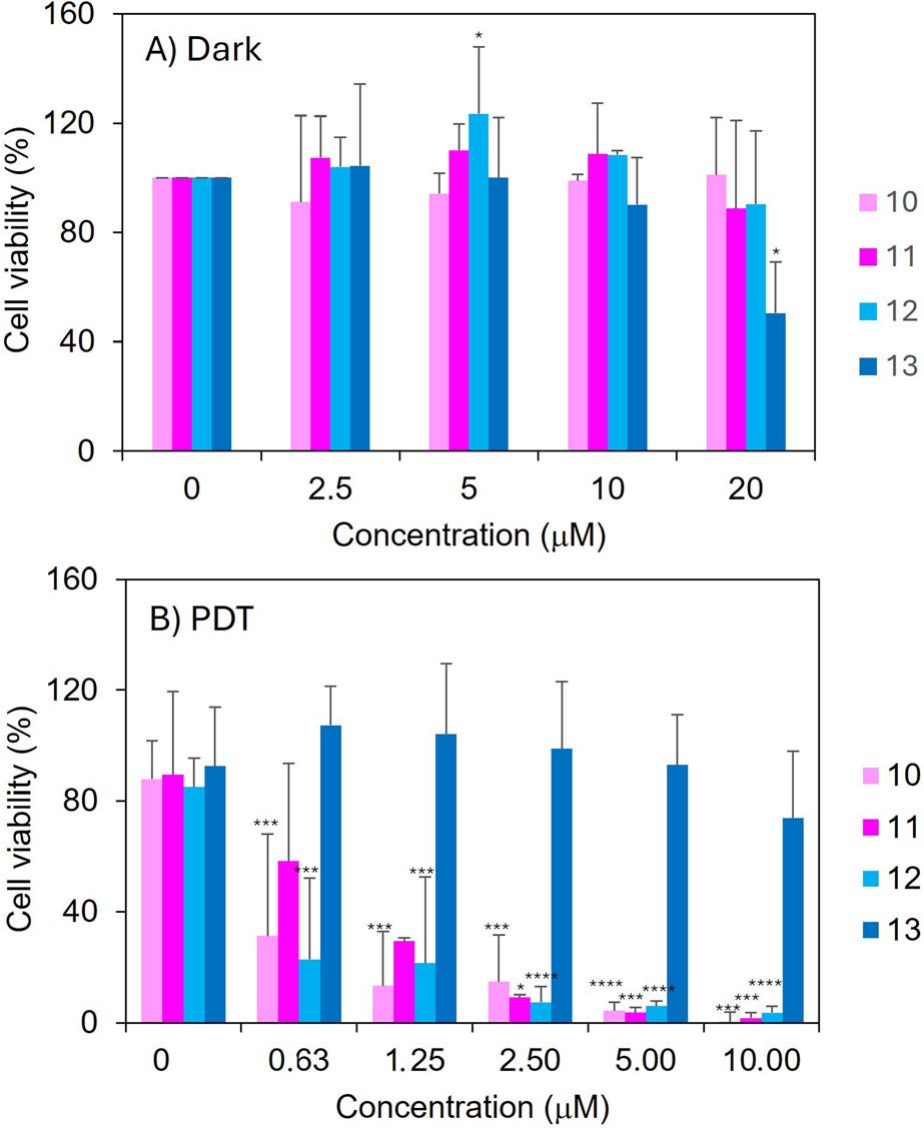
Viability of HeLa cells treated with compounds 10–13 at different concentration, measured (A) under non-irradiated condition (“dark”) and (B) after irradiation (“PDT”) for 500 seconds, with a fluence of 5 J cm^−2^ and an irradiance of 10 mW cm^−2^ at 420 nm. Data are presented as mean plus standard deviation. Statistical analysis was conducted using a one-way ANOVA followed by Dunnett's multiple comparison test. Asterisks (*) indicates statistical significance relative to the untreated control (0 µM): **** = *p* < 0.0001; *** = *p* < 0.001; ** = *p* < 0.01; * = *p* < 0.05. Values without asterisks are not significantly different from the untreated control.

The potential of the glycofullerene–corrole conjugates 12 and 13 to act as a PS was evaluated in the HeLa cell line and compared with the efficacy of the precursor corroles 10 and 11. Dark cytotoxicity was initially assessed by measuring the cell viability after 12 h of incubation with concentrations ranging from 0 to 20 µM. As shown in Fig. 3A, with the exception of glycofullerene–corrole conjugate 13, all compounds show a high cell viability up to 20 µM. A dark toxicity significantly different from the control (non-treated cells) is only observed for conjugate 13 at concentrations above 10 µM. Thus, this concentration was set as the higher limit in the studies of photoinduced cytotoxicity. The phototoxicity was assessed in a concentration range below 10 µM (0.6–10 µM), under blue (420 nm) light irradiation at doses of 1, 5 and 10 J cm^−2^ for 100, 500 and 1000 seconds. The phototoxicity of all derivatives (10–13) at total light dose of 5 J cm^−2^ is compiled in Fig. 3B. Fig. S37 displays the light dose dependence of cell viability for each compound at different concentrations. Except for glycofullerene–corrole conjugate 13, all the compounds tested showed a clear and systematic decrease of the cell viability with the increase in the irradiation light dose, even at the lowest tested concentration (Fig. S37). Compounds 10, 11, and 12 demonstrated a strong photosensitizing effect, reducing cell viability to below 20% under a total light dose of 10 J cm^−2^, even at the lowest concentration tested. Conversely, conjugate 13 does not show a significant decrease of cell viability even at the high concentrations (10 µM) and at the highest light dose (10 J cm^−2^) tested. The observed photosensitizing effect correlates well with the ^1^O_2_ generation yield measured in solution. The weak photosensitizing effect of 13 could be anticipated by its lower ^1^O_2_ generation quantum yield.

Compounds 10, 11, and 12 exhibit IC_50_ values of 4.16 µM, 4.96 µM, and 4.42 µM, respectively, under irradiation at 1 J cm^−2^ (Table S1). Under a higher light dose of 5 J cm^−2^, their IC_50_ values are below 1.0 µM.

#### 2.2.2 Fluorescence microscopy

**Fig. 4:**
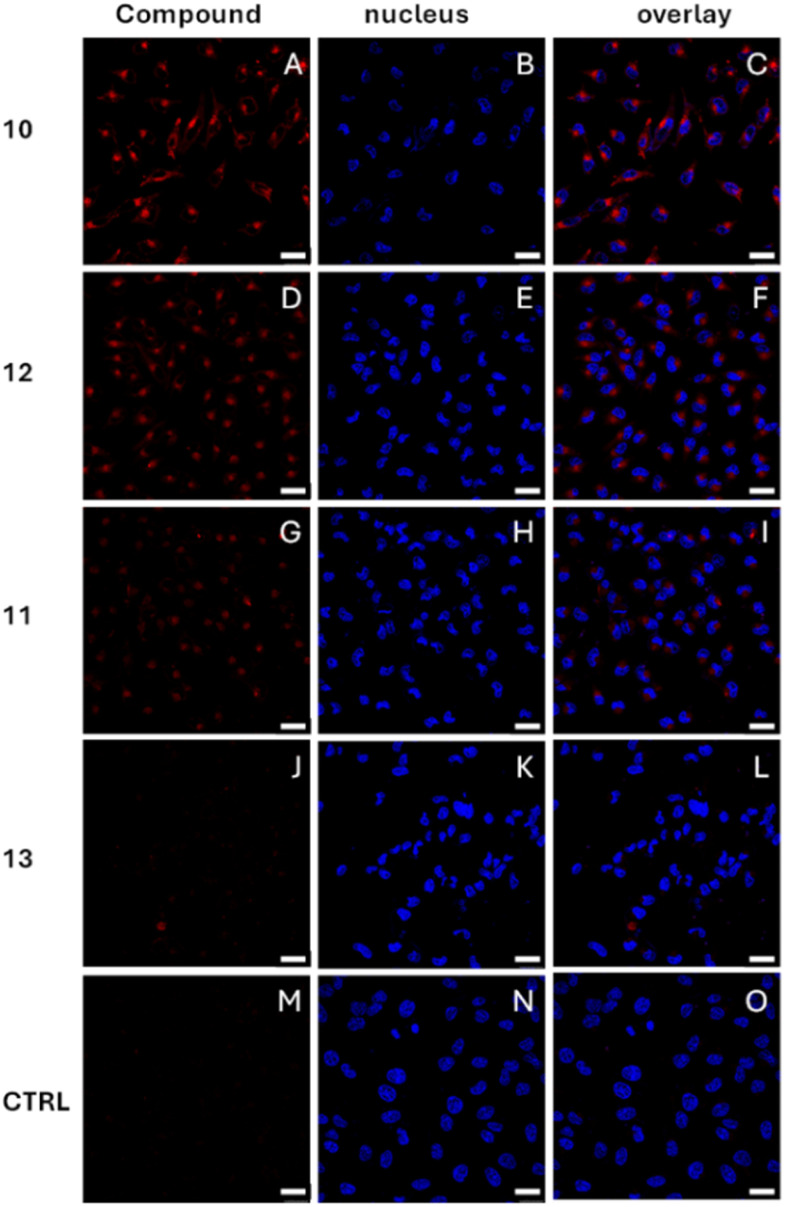
Fluorescence image of the HeLa cells incubated overnight with 10 µM of compounds 10 (A–C), 11 (G–I), 12 (D–F) and 13 (J–L). The red channel shows emission of the corrole deriva ves (exc. 514 nm/em 600–700 nm), the blue channel shows emission of the commercial nuclear stain (exc. 780 nm/em. 420–500 nm) and the rightmost panel shows the overlay of both channels. The scale bar is 25 µm. A control experiment (CTRL (M-O)) is also included, in which the cells were incubated without any PS.

The cellular uptake and distribution of the compounds was also evaluated in HeLa cells by confocal and multiphoton fluorescence microscopy. Fig. 4 shows the fluorescence microscopy images of the HeLa cells recorded upon incubation overnight with 10 µM of compounds 10–13 in the incubation media. The fluorescence of the compounds, (red channel in Fig. 4) was excited at 514 nm and collected within the 600–700 nm range. A commercial stain (Hoescht) was used to label the nucleus. The emission of the nuclear stain, shown in the blue channel in Fig. 4, was excited by multiphoton excitation at 780 nm and collected within the 420–500 nm range. The emission of the corroles 10 and 11, and that of the conjugate 12 are clearly observed inside the cells, localized within the cytoplasmic compartment. The lack of overlap between the red emission of the corrole derivatives and the blue emission of the nuclear stain indicates that the compounds do not accumulate in the nucleus. Fig. S38 shows that all the compounds exhibit a predominantly diffuse cytoplasmic distribution together with more intense punctate structures, consistent with accumulation in lysosomal compartments. The average emission intensity of corrole 10 (Fig. 4A) and the corresponding glycofullerene conjugate 12 (Fig. 4D) inside the cells is similar, while the emission intensity of corrole 11 (Fig. 4G) is lower by a factor of 2. The emission of conjugate 13 (Fig. 4J) is barely distinguishable from the autofluorescence of the control in the bottom line (Fig. 4M). Considering that the *Φ*_F_ of conjugate 12 is lower than that of corrole 10, the observation of a similar emission intensity within the cells for both compounds suggests that the cellular uptake of conjugate 12 is more efficient than that of its corresponding corrole 10. A weaker emission of corrole 11 inside the cells as compared with corrole 10, both with similar *Φ*_F_, suggest that the former is less efficiently internalized by the cells.

## 3. Experimental

### 3.1 Materials and methods

Reagents and solvents were obtained from commercial suppliers and used without further purification. All work-up and purification procedures were carried out with HPLC-grade solvents in air. Microwave reactions were performed in an Anton-Parr Monowave 300 microwave reactor. Flash chromatography was performed using Scharlab silica gel (230–400 mesh, 0.040–0.063 mm) and for gel filtration Sephadex G-25 or G-50 were purchased from Cytiva. Analytical thin layer chromatography (TLC) was performed using E. Merck silica gel 60 F_254_ precoated plates (0.25 mm). The developed chromatogram was analyzed by UV lamp (*λ* = 254 nm) charring with potassium permanganate as development reagent.

^1^H, ^13^C and ^19^F NMR spectra were performed on a Bruker AVIII HD 300 MHz BACS-60 (^1^H: 300 MHz, ^13^C: 75 MHz), Bruker NEO 500 MHz (^1^H: 500 MHz, ^19^F: 471 MHz) and Bruker AVIII 700 MHz (^1^H: 700 MHz, ^13^C: 176 MHz) at 298 K using partially deuterated solvents as internal standards. Coupling constants (*J*) are expressed in Hertz (Hz) and chemical shifts (*δ*) in parts per million (ppm) relative to the solvent. Data are reported as follows: s = singlet, t = triplet, q = quartet, m = multiplet. IR spectra (cm^−1^) were recorded on a Spectrum 3™ FT-IR spectrometer. Mass analyses were carried out in a MALDI TOF/TOF Bruker Ultraflex using *trans*-2-[3-(4-^*t*^butylphenyl)-2-methyl-2-propenylidene]malononitrile (DCTB) or 1,8-dihydroxi-9,10-dihydroanthracen-9-one (dithranol) as matrixes. Hydrodynamic diameter measurements were acquired on a Dynamic Light Scattering (DLS) Zetasizer Nano ZS Zen 3600 (Malvern), working with an He–Ne laser operating at *λ* = 633 nm. The samples were recorded with a scattering angle of 173°. Measurements were made in a 1.0 cm path-length quartz cell. Solution samples were filtered through nylon Acrodisc syringe filters (Pall Life Sciences) with a 0.2 µm pore size.

### 3.2 Photophysical measurements

The UV-vis absorption spectra were recorded with a JASCO V-540 spectrophotometer. The photoluminescence spectra were obtained on a Horiba Jobin Yvon Fluorolog 3-22 spectrofluorimeter equipped with a 450 W xenon lamp.

Fluorescence microscopy images were acquired using a Leica TCS-SP5 laser scanning confocal fluorescence microscope. The system is equipped with two continuous-wave lasers: an argon laser providing excitation lines at 458, 488, 496, and 514 nm, and a HeNe laser offering excitation at 633 nm. Additionally, a Ti:Sapphire pulsed laser (Mai Tai, Spectra-Physics) delivers tunable excitation in the 730–990 nm range, with a pulse width of 100 fs and a repetition rate of 82 MHz.

### 3.3 Synthesis

Compounds 1 (ref. 47) and 2 (ref. 48) were previously reported, but a different synthetic methodology was used in this work. Compounds 3,^48^4,^49^6,^50^ and 9 (ref. 51) were prepared according to previously reported procedures (Schemes S1 and S2).

#### 3.3.1 Compound 1

Triphenylphosphine (1.21 g, 4.62 mmol) dissolved in dry CH_2_Cl_2_ (2 mL) was added dropwise to a solution of pentaethylene glycol (3 g, 12.60 mmol) and CBr_4_ (1.39 g, 4.20 mmol) in dry CH_2_Cl_2_ (50 mL) at 0 °C under argon atmosphere. After 30 min, the mixture was allowed to warm slowly to room temperature and stirred for 3 h. After that time, the solvent was evaporated to dryness and the resulting crude was purified by column chromatography (SiO_2_, AcOEt) to obtain 1 as a colourless oil (462 mg, 36%) (Fig. S1 and S2).

^1^H NMR (300 MHz, CDCl_3_) *δ* (ppm) = 3.81 (t, ^3^*J*_*H*,*H*_ = 6.3 Hz, 2H; C*H*_2_CH_2_Br), 3.75–3.70 (m, 2H; HOCH_2_C*H*_2_O), 3.70–3.64 (m, 12H; C*H*_2_O), 3.63–3.59 (m, 2H; HOC*H*_2_CH_2_O), 3.47 (t, ^3^*J*_*H*,*H*_ = 6.3 Hz, 2H; C*H*_2_Br), 2.34 (br s, 1H; O*H*).

^13^C NMR (75 MHz, CDCl_3_) *δ* (ppm) = 72.7 (OH*C*H_2_CH_2_O), 71.3 (*C*H_2_CH_2_Br), 70.7, 70.6 (*C*H_2_O), 61.8 (OHCH_2_*C*H_2_O), 30.4 (*C*H_2_Br).

#### 3.3.2 Compound 2

Ethyl malonyl chloride (0.13 mL, 1.05 mmol) was added dropwise to a solution of 1 (0.35 g, 1.15 mmol), Et_3_N (0.16 mL, 1.15 mmol) and DMAP (6 mg, 0.053 mmol) in dry CH_2_Cl_2_ (10 mL) at 0 °C under argon atmosphere. After 30 min, the mixture was allowed to warm slowly to room temperature and stirred overnight. Then, the solution was washed with HCl 1 M (20 mL) and brine (20 mL). The organic layer was dried over anhydrous MgSO_4_, filtered and concentrated. The resulting crude was purified by column chromatography (SiO_2_, AcOEt) to obtain 2 as a colourless oil (402 mg, 92%) (Fig. S3 and S4).

^1^H NMR (300 MHz, CDCl_3_) *δ* (ppm) = 4.31–4.24 (m, 2H; COOC*H*_2_CH_2_O), 4.18 (q, ^3^*J*_*H*,*H*_ = 7.2 Hz, 2H; C*H*_2_CH_3_), 3.78 (t, ^3^*J*_*H*,*H*_ = 6.3 Hz, 2H; C*H*_2_CH_2_Br), 3.71–3.67 (m, 2H; COOCH_2_C*H*_2_O), 3.66–3.60 (m, 12H; C*H*_2_O), 3.45 (t, ^3^*J*_*H*,*H*_ = 6.3 Hz, 2H; C*H*_2_Br), 3.38 (s, 2H; COC*H*_2_CO), 1.26 (t, ^3^*J*_*H*,*H*_ = 7.2 Hz, 3H; C*H*_3_).

^13^C NMR (75 MHz, CDCl_3_) *δ* (ppm) = 166.7, 166.5 (*C*O), 71.3 (*C*H_2_CH_2_Br), 70.7, 70.6 (*C*H_2_O), 68.9 (COOCH_2_*C*H_2_O), 64.6 (COO*C*H_2_CH_2_O), 61.6 (*C*H_2_CH_3_), 41.6 (CO*C*H_2_CO), 30.4 (*C*H_2_Br), 14.2 (*C*H_3_).

#### 3.3.3 Compound 5

DBU (1.35 mL, 9.00 mmol) was added dropwise to a solution of fullerene 3(510 mg, 0.45 mmol),^48^ bis(pent-4-yn-1-yl) malonate 4 (1.06 g, 4.50 mmol) and CBr_4_ (11.94 g, 36.00 mmol) in dry toluene (165 mL) under argon atmosphere. The mixture was stirred at room temperature for 72 h. After that time, the reaction mixture was washed with Na_2_S_2_O_3_ sat. (100 mL), HCl 1 M (2 × 100 mL), water (100 mL) and brine (100 mL). The organic layer was dried over anhydrous MgSO_4_, filtered and concentrated under vacuum. The crude was purified by column chromatography (SiO_2_, DCM → DCM/Acetone gradient) to obtain 5 as a dark red solid (737 mg, 71%) (Fig. S5–S7).

^1^H NMR (700 MHz, CDCl_3_) *δ* (ppm) = 4.60–4.11 (m, 24H; COOC*H*_2_), 3.76 (t, ^3^*J*_*H*,*H*_ = 6.3 Hz, 2H; C*H*_2_CH_2_Br), 3.74–3.66 (m, 2H; COOCH_2_C*H*_2_O), 3.65–3.54 (m, 12H; C*H*_2_O_PEG_), 3.43 (t, ^3^*J*_*H*,*H*_ = 6.3 Hz, 2H; C*H*_2_Br), 2.45–2.14 (m, 20H; COOCH_2_CH_2_C*H*_2_), 2.06–1.59 (m, 30H; COOCH_2_C*H*_2_CH_2_ and C≡C*H*), 1.40–1.20 (m, 3H; C*H*_3_).

^13^C NMR (176 MHz, CDCl_3_) *δ* (ppm) = 163.7 (*C*O), 145.8, 141.0 (*C*_sp^2^ fullerene_), 82.5 (*C*≡CH), 78.4 (C≡*C*H), 71.2 (*C*H_2_CH_2_Br), 70.6, 70.5 (*C*H_2_O_PEG_), 69.8 (C≡*C*H), 69.0 (*C*_sp^3^ fullerene_), 68.6 (COOCH_2_*C*H_2_O), 65.8 (COO*C*H_2_CH_2_O), 65.4 (COO*C*H_2_CH_2_CH_2_), 63.0 (*C*H_2_CH_3_), 45.3, 39.4 (*C*_q bridge_), 30.4 (*C*H_2_Br), 27.1 (COOCH_2_*C*H_2_CH_2_), 16.4, 15.1 (COOCH_2_CH_2_*C*H_2_), 14.1 (*C*H_3_).

FTIR-ATR (*ν* cm^−1^): 3293 (C≡C–H), 2958 (C–H), 1739 (C=O).

#### 3.3.4 Compound 7

A mixture of 5 (202 mg, 0.088 mmol), the azido sugar derivative 6 (393 mg, 1.56 mmol)^49^ CuBr·S(CH_3_)_2_ (91 mg, 0.44 mmol), sodium ascorbate (131 mg, 0.66 mmol) and a piece of copper metal wire in DMSO (1 mL) was deoxygenated and kept under argon with vigorous stirring for 72 h. The crude reaction was filtered through a Quadrasil^®^ mercaptopropyl column. CH_3_OH was added to the mixture and the precipitate was centrifuged and extensively washed with CH_3_OH, then dried under high vacuum to obtain 7 as a brown solid (386 mg, 91%) (Fig. S8–S10).

^1^H NMR (700 MHz, DMSO-*d*_6_) *δ* (ppm) = 8.05 (s, 10H; C*H*_triazole_), 5.10 (s, 10H; O*H*), 4.99 (s, 10H; O*H*), 4.94 (s, 10H; O*H*), 4.61–4.48 (m, 30H; OCH_2_C*H*_2_N_glu_ and O*H*), 4.44–4.25 (m, 22H; C*H*_2_CH_3_ and COOC*H*_2_CH_2_CH_2_), 4.24–4.18 (m, 12H; C*H*_1glu_ and COOC*H*_2_CH_2_O), 4.11–4.05 (m, 10H; OC*H*_2_CH_2_N_glu_), 3.93–3.85 (m, 10H; OC*H*_2_CH_2_N_glu_), 3.70–3.64 (m, 12H; C*H*_2_OH_glu_ and C*H*_2_O_PEG_), 3.56–3.53 (m, 4H; C*H*_2_Br and C*H*_2_O_PEG_), 3.52–3.46 (m, 10H; C*H*_2_O_PEG_), 3.45–3.40 (m, 12H; C*H*_2_OH_glu_ and C*H*_2_O_PEG_), 3.16–3.10 (m, 20H; C*H*_glu_), 3.06–3.01 (m, 10H; C*H*_glu_), 2.99–2.93 (m, 10H; C*H*_glu_), 2.83–2.59 (m, 20H; COOCH_2_CH_2_C*H*_2_), 2.10–1.84 (m, 20H; COOCH_2_C*H*_2_CH_2_), 1.28–1.13 (m, 3H; C*H*_3_).

^13^C NMR (176 MHz, DMSO-*d*_6_) *δ* (ppm) = 162.8 (*C*O), 145.9 (*C*_triazole_), 145.0, 140.7 (*C*_sp^2^ fullerene_), 123.5 (*C*H_triazole_), 102.8 (*C*H_1glu_), 76.9, 76.5, 73.2 (*C*H_glu_), 70.3 (*C*H_2_O_PEG_), 70.0 (*C*H_glu_), 69.7, 69.5 (*C*H_2_O_PEG_), 68.7 (*C*_sp^3^ fullerene_), 67.9 (COO*C*H_2_CH_2_O), 67.1 (O*C*H_2_CH_2_N_glu_), 66.6 (COO*C*H_2_CH_2_CH_2_), 63.3 (*C*H_2_CH_3_), 61.0 (*C*H_2_OH_glu_), 49.9 (OCH_2_*C*H_2_N_glu_), 45.5 (*C*_q bridge_), 32.2 (*C*H_2_Br), 27.6 (COOCH_2_*C*H_2_CH_2_), 21.5 (COOCH_2_CH_2_*C*H_2_), 13.7 (*C*H_3_).

IR (KBr) (*ν* cm^−1^): 3401 (OH), 2920 (C–H), 1741 (C=O).

#### 3.3.5 Compound 8

A mixture of 7 (150 mg, 0.031 mmol) and NaN_3_ (20 mg, 0.31 mmol) in DMSO-*d*_6_ (0.8 mL) was heated for 90 min at 55 °C under microwave irradiation. The crude was purified by size-exclusion chromatography (Sephadex G-25 H_2_O/CH_3_OH 9 : 1) to obtain 8 as a brown glassy solid (138 mg, 93%) (Fig. S11–S13).

^1^H NMR (700 MHz, DMSO-*d*_6_) *δ* (ppm) = 7.91 (s, 10H; C*H*_triazole_), 5.10 (s, 10H; O*H*), 4.98 (s, 10H; O*H*), 4.93 (s, 10H; O*H*), 4.53 (s, 10H; O*H*), 4.52–4.44 (m, 20H; OCH_2_C*H*_2_N), 4.42–4.24 (m, 24H; C*H*_2_CH_3_, COOC*H*_2_CH_2_CH_2_ and COOC*H*_2_CH_2_O), 4.21 (d, ^3^*J*_*H*,*H*_ = 7.7 Hz, 10H; C*H*_1glu_), 4.08–4.03 (m, 10H; OC*H*_2_CH_2_N), 3.89–3.83 (m, 10H; OC*H*_2_CH_2_N), 3.70–3.63 (m, 12H; C*H*_2_OH_glu_ and C*H*_2_O_PEG_), 3.58–3.56 (m, 2H; C*H*_2_O_PEG_), 3.53–3.46 (m, 10H; C*H*_2_O_PEG_), 3.45–3.41 (m, 12H; C*H*_2_OH_glu_ and C*H*_2_O_PEG_), 3.36 (m, 2H; C*H*_2_N_3_ overlaps with water), 3.16–3.10 (m, 20H; C*H*_glu_), 3.06–3.02 (m, 10H; C*H*_glu_), 2.99–2.93 (m, 10H; C*H*_glu_), 2.75–2.58 (m, 20H; COOCH_2_CH_2_C*H*_2_), 2.09–1.87 (m, 20H; COOCH_2_C*H*_2_CH_2_), 1.28–1.15 (m, 3H; C*H*_3_).

^13^C NMR (176 MHz, DMSO-*d*_6_) *δ* (ppm) = 162.9 (*C*O), 145.6 (*C*_triazole_), 145.1, 140.7 (*C*_sp^2^ fullerene_), 122.8 (*C*H_triazole_), 102.8 (*C*H_1glu_), 77.0, 76.6, 73.3, 70.0 (*C*H_glu_), 69.8, 69.7, 69.3 (*C*H_2_O_PEG_), 68.7 (*C*_sp^3^ fullerene_), 68.0 (COO*C*H_2_CH_2_O), 67.3 (O*C*H_2_CH_2_N_glu_), 66.7 (COO*C*H_2_CH_2_CH_2_), 63.4 (*C*H_2_CH_3_), 61.1 (*C*H_2_OH_glu_), 50.0 (*C*H_2_N_3_), 49.5 (OCH_2_*C*H_2_N_glu_), 45.6 (*C*_q bridge_), 27.7 (COOCH_2_*C*H_2_CH_2_), 21.4 (COOCH_2_CH_2_*C*H_2_), 13.8 (*C*H_3_).

IR (KBr) (*ν* cm^−1^): 3401 (OH), 2921 (C–H), 2108 (N_3_), 1741 (C=O).

#### 3.3.6 Compound 10

Propargyl alcohol (2.2 µL, 0.038 mmol) was added to a solution of Ga(iii) corrole complex 9 (30 mg, 0.032 mmol) and K_2_CO_3_ (22 mg, 0.16 mmol) in DMSO (5 mL). The mixture was heated at 100 °C for 3 h. After that time, CHCl_3_ (100 mL) was added and the organic layer was washed with water (50 mL), dried over anhydrous MgSO_4_, filtered and concentrated. The resulting crude product was purified by column chromatography (SiO_2_, Hexane/AcOEt/pyridine 3 : 1 : 0.02) to obtain the monosubstituted derivatives 10 as a fuchsia solid (5 mg, 16%). These derivatives were obtained as a 2 : 1 mixture of the inseparable regioisomers 10a and 10b (Fig. S14–S17).

^1^H NMR (500 MHz, CD_2_Cl_2_) *δ* (ppm) = 9.28 [d, ^3^*J*_*H*,*H*_ = 4.0 Hz, 3H; C*H*_β_ (2H, 10a; 1H, ½ 10b)], 8.95 (d, ^3^*J*_*H*,*H*_ = 4.6 Hz, 1H; C*H*_β_, ½ 10b), 8.91 (d, ^3^*J*_*H*,*H*_ = 4.3 Hz, 2H; C*H*_β_, 10a), 8.88 (d, ^3^*J*_*H*,*H*_ = 4.0 Hz, 1H; C*H*_β_, ½ 10b), 8.84 (d, ^3^*J*_*H*,*H*_ = 4.0 Hz, 2H; C*H*_β_, 10a), 8.74 (d, ^3^*J*_*H*,*H*_ = 4.6 Hz, 1H; C*H*_β_, ½ 10b), 8.70 (d, ^3^*J*_*H*,*H*_ = 4.3 Hz, 2H; C*H*_β_, 10a), 6.69–6.61 [m, 1.5H; C*H*_*para* py_ (1H, 10a; 0.5H, ½ 10b)], 5.89–5.80 [m, 3H; C*H*_*meta* py_ (2H, 10a; 1H, ½ 10b)], 5.20 (d, ^3^*J*_*H*,*H*_ = 2.4 Hz, 2H; OC*H*_2_, 10a), 5.18 (d, ^3^*J*_*H*,*H*_ = 2.4 Hz, 1H; OC*H*_2_, ½ 10b), 2.98–2.86 [m, 4H; C*H*_*ortho* py_ (2H, 10a; 1H, ½ 10b) and C≡C*H*, 10a], 2.85 (t, ^3^*J*_*H*,*H*_ = 2.4 Hz, 0.5H; C≡C*H*, ½ 10b).

^19^F{^1^H} NMR (471 MHz, CD_2_Cl_2_) *δ* (ppm) = −138.86–−139.33 [m; *F*_*ortho*_ (10-*F*_*ortho*_, 15-*F*_*ortho*_, 10a; 5-*F*_*ortho*_, 15-*F*_*ortho*_, 10b)], −140.78 [dd, ^3^*J*_*F*,*F*_ = 23.8, 8.9 Hz; *F*_*ortho*_, (5-*F*_*ortho*_, 10a; 10-*F*_*ortho*_, 10b)], −155.34–−155.39 (m; *F*_*para*_, 10a), −155.64 (t, ^3^*J*_*F*,*F*_ = 20.6 Hz; *F*_*para*_, 10b), −157.46 (dd, ^3^*J*_*F*,*F*_ = 23.1, 8.6 Hz; 5-*F*_*meta*_, 10a), −157.64 (dd, ^3^*J*_*F*,*F*_ = 24.0, 8.3 Hz; 10-*F*_*meta*_, 10b), −163.73–−163.93 (m; 10-*F*_*meta*_ and 15-*F*_*meta*_, 10a), −164.02 (dd, ^3^*J*_*F*,*F*_ = 25.0, 5.0 Hz; 5-*F*_*meta*_ and 15-*F*_*meta*_, 10b).

MALDI-TOF: *m*/*z* calcd for C_40_H_11_F_14_GaN_4_O: 898.0000, found: 898.0750 [M^+^].

#### 3.3.7 Compound 11

Propargyl alcohol (2.6 µL, 0.044 mmol) and NaH (8 mg, 0.33 mmol) were dissolved in dry THF (5 mL) and heated to reflux for 30 min. Then, a solution of 9 (10 mg, 0.011 mmol) in dry THF was added and the mixture was heated to reflux for 1 h. After that time, CH_2_Cl_2_ (50 mL) was added and the organic layer was washed with water (50 mL), dried over anhydrous MgSO_4_, filtered and concentrated. The resulting crude was purified by column chromatography (SiO_2_, Hexane/AcOEt/pyridine 3 : 1 : 0.02) to obtain 11 as a fuchsia solid (6 mg, 52%) (Fig. S18–S21).

^1^H NMR (500 MHz, CD_2_Cl_2_) *δ* (ppm) = 9.27 (d, ^3^*J*_*H*,*H*_ = 4.0 Hz, 2H; C*H*_β_), 8.94 (d, ^3^*J*_*H*,*H*_ = 4.5 Hz, 2H; C*H*_β_), 8.86 (d, ^3^*J*_*H*,*H*_ = 4.0 Hz, 2H; C*H*_β_), 8.72 (d, ^3^*J*_*H*,*H*_ = 4.5 Hz, 2H; C*H*_β_), 6.70–6.63 (m, 1H; C*H*_*para* py_), 5.88–5.84 (m, 2H; C*H*_*meta* py_), 5.20 (d, ^3^*J*_*H*,*H*_ = 2.4 Hz, 4H; OC*H*_2_), 5.18 (d, ^3^*J*_*H*,*H*_ = 2.4 Hz, 2H; OC*H*_2_), 2.94 (d, ^3^*J*_*H*,*H*_ = 5.4 Hz, 2H; C*H*_*ortho* py_), 2.87 (t, ^3^*J*_*H*,*H*_ = 2.4 Hz, 2H; C≡C*H*), 2.85 (t, ^3^*J*_*H*,*H*_ = 2.4 Hz, 1H; C≡C*H*).

^19^F{^1^H} NMR (471 MHz, CD_2_Cl_2_) *δ* (ppm) = −140.69–−140.83 (m; *F*_*ortho*_), −157.52 (dd, ^3^*J*_*F*,*F*_ = 23.6, 8.8 Hz; *F*_*meta*_), −158.71 (dd, ^3^*J*_*F*,*F*_ = 24.2, 9.0 Hz; *F*_*meta*_).

MALDI-TOF: *m*/*z* calcd for C_51_H_22_F_12_GaN_5_O_3_: 1049.0787, found: 1049.1874 [M]^+.^.

FTIR-ATR (*ν* cm^−1^): 3303 (C≡C–H), 2929 (C–H), 1489 (C=N).

#### 3.3.8 Compound 12

A mixture of glycofullerene 8 (82 mg, 0.018 mmol), corrole 10 (13.4 mg, 0.014 mmol), CuBr·S(CH_3_)_2_ (3 mg, 0.014 mmol), sodium ascorbate (4 mg, 0.021 mmol) and a piece of copper metal wire in DMSO (1.5 mL) was deoxygenated and kept under argon with vigorous stirring for 48 h. The crude reaction was filtered through a Quadrasil^®^ mercaptopropyl column. Then, it was purified by size-exclusion chromatography (Sephadex G-50 H_2_O/CH_3_OH 9 : 1) to obtain 12 as a violet solid (60 mg, 74%) (Fig. S22–S25).

The product was obtained as a mixture of isomers indistinguishable by ^1^H and ^13^C NMR. For simplicity, only the major isomer (12a) is represented in Fig. 1.

^1^H NMR (700 MHz, DMSO-*d*_6_) *δ* (ppm) = 9.36–8.52 (m, 8H; C*H*_β corrole_), 8.48 (s, 1H; C*H*_triazole_), 7.91 (s, 10H; C*H*_triazole_), 5.66 (m, 2H; OC*H*_2 corrole_), 5.12 (s, 10H; O*H*), 5.00 (s, 10H; O*H*), 4.95 (s, 10H; O*H*), 4.67–4.64 (m, 2H; OCH_2_C*H*_2_N_PEG_), 4.55 (s, 10H; O*H*), 4.52–4.46 (m, 20H; OCH_2_C*H*_2_N_glu_), 4.41–4.27 (m, 24H; C*H*_2_CH_3_, COOC*H*_2_CH_2_CH_2_ and COOC*H*_2_CH_2_O), 4.21 (d, ^3^*J*_*H*,*H*_ = 7.7 Hz, 10H; C*H*_1glu_), 4.08–4.04 (m, 10H; OC*H*_2_CH_2_N_glu_), 3.89–3.84 (m, 12H; OC*H*_2_CH_2_N_glu_ and C*H*_2_O_PEG_), 3.69–3.65 (m, 12H; C*H*_2_OH_glu_ and C*H*_2_O_PEG_), 3.56–3.54 (m, 2H; C*H*_2_O_PEG_), 3.48–3.42 (m, 22H; C*H*_2_OH_glu_ and C*H*_2_O_PEG_), 3.15–3.11 (m, 20H; C*H*_glu_), 3.06–3.03 (m, 10H; C*H*_glu_), 2.98–2.95 (m, 10H; C*H*_glu_), 2.69–2.58 (m, 20H; COOCH_2_CH_2_C*H*_2_), 2.02–1.90 (m, 20H; COOCH_2_C*H*_2_CH_2_), 1.25–1.15 (m, 3H; C*H*_3_).

^13^C NMR (176 MHz, DMSO-*d*_6_) *δ* (ppm) = 162.9 (*C*O), 145.6 (*C*_triazole_), 145.1 (*C*_sp^2^ fullerene_), 141.8 (*C*_corrole_), 140.8 (*C*_sp^2^ fullerene_), 138.1, 136.7 (*C*_corrole_), 125.9, 122.8 (*C*H_triazole_), 102.9 (*C*H_1glu_), 77.0, 76.6, 73.3, 70.0 (*C*H_glu_), 69.7 (*C*H_2_O_PEG_), 68.9 (*C*_sp^3^ fullerene_ and O*C*H_2 corrole_), 68.0 (COO*C*H_2_CH_2_O), 67.4 (O*C*H_2_CH_2_N_glu_), 66.8 (COO*C*H_2_CH_2_CH_2_), 63.4 (*C*H_2_CH_3_), 61.1 (*C*H_2_OH_glu_), 49.5 (OCH_2_*C*H_2_N), 45.7 (*C*_q bridge_), 27.7 (COOCH_2_*C*H_2_CH_2_), 21.5 (COOCH_2_CH_2_*C*H_2_), 13.8 (*C*H_3_).

^19^F{^1^H} NMR (471 MHz, DMSO-*d*_6_) *δ* (ppm) = −139.27 to −140.25 [m; *F*_*ortho*_ (10-*F*_*ortho*_, 15-*F*_*ortho*_, 12a; 5-*F*_*ortho*_, 15-*F*_*ortho*_, 12b)], −140.81 to −141.50 [m; *F*_*ortho*_ (5-*F*_*ortho*_, 12a; 10-*F*_*ortho*_, 12b)], −155.37 to −156.21 (m; *F*_*para*_), −156.59 to −157.60 [m; *F*_*meta*_ (5-*F*_*meta*_, 12a; 10-*F*_*meta*_, 12b)], −163.17 to −164.55 [m; *F*_*meta*_ (10-*F*_*meta*_ and 15-*F*_*meta*_, 12a; 5-*F*_*meta*_ and 15-*F*_*meta*_, 12b)].

IR (KBr) (*ν* cm^−1^): 3414 (OH), 2926 (C–H), 1740 (C=O).

#### 3.3.9 Compound 13

A mixture of glycofullerene 8 (192 mg, 0.040 mmol), corrole 11 (11 mg, 0.011 mmol), CuBr·S(CH_3_)_2_ (11 mg, 0.055 mmol), sodium ascorbate (16 mg, 0.083 mmol) and a piece of copper metal wire in DMSO (1.5 mL) was deoxygenated and kept under argon with vigorous stirring for 72 h. The crude reaction was filtered through a Quadrasil^®^ mercaptopropyl column. Then, it was purified by size-exclusion chromatography (Sephadex G-50 H_2_O/CH_3_OH 9 : 1) to obtain 13 as a purple solid (137 mg, 81%) (Fig. S26–29).

^1^H NMR (700 MHz, DMSO-*d*_6_) *δ* (ppm) = 9.34–8.60 (m, 8H; C*H*_β corrole_), 8.48 (s, 3H; C*H*_triazole_), 7.91 (s, 30H; C*H*_triazole_), 5.66 (s, 6H; OC*H*_2 corrole_), 5.11 (s, 30H; O*H*), 4.99 (s, 30H; O*H*), 4.94 (s, 30H; O*H*), 4.67–4.63 (m, 6H; OCH_2_C*H*_2_N_PEG_), 4.54 (s, 30H; O*H*), 4.51–4.45 (m, 60H; OCH_2_C*H*_2_N_glu_), 4.42–4.27 (m, 72H; C*H*_2_CH_3_, COOC*H*_2_CH_2_CH_2_ and COOC*H*_2_CH_2_O), 4.21 (d, ^3^*J*_*H*,*H*_ = 8.0 Hz, 30H; C*H*_1glu_), 4.08–4.04 (m, 30H; OC*H*_2_CH_2_N_glu_), 3.90–3.84 (m, 36H; OC*H*_2_CH_2_N_glu_ and C*H*_2_O_PEG_), 3.69–3.64 (m, 36H; C*H*_2_OH_glu_ and C*H*_2_O_PEG_), 3.57–3.55 (m, 6H; C*H*_2_O_PEG_), 3.49–3.41 (m, 66H; C*H*_2_OH_glu_ and C*H*_2_O_PEG_), 3.15–3.10 (m, 60H; C*H*_glu_), 3.06–3.02 (m, 30H; C*H*_glu_), 2.98–2.94 (m, 30H; C*H*_glu_), 2.72–2.59 (m, 60H; COOCH_2_CH_2_C*H*_2_), 2.04–1.89 (m, 60H; COOCH_2_C*H*_2_CH_2_), 1.28–1.16 (m, 9H; C*H*_3_).

^13^C NMR (176 MHz, DMSO-*d*_6_) *δ* (ppm) = 162.9 (*C*O), 145.6 (*C*_triazole_), 145.1 (*C*_sp^2^ fullerene_), 141.9 (*C*_corrole_), 140.8 (*C*_sp^2^ fullerene_), 136.6 (*C*_corrole_), 125.9, 122.8 (*C*H_triazole_), 102.8 (*C*H_1glu_), 77.0, 76.6, 73.3, 70.0 (*C*H_glu_), 69.7 (*C*H_2_O_PEG_), 68.8 (*C*_sp^3^ fullerene_), 68.0 (COO*C*H_2_CH_2_O), 67.3 (O*C*H_2_CH_2_N_glu_ and O*C*H_2 corrole_), 66.7 (COO*C*H_2_CH_2_CH_2_), 63.4 (*C*H_2_CH_3_), 61.1 (*C*H_2_OH_glu_), 49.5 (OCH_2_*C*H_2_N_PEG_ and OCH_2_*C*H_2_N_glu_), 45.6 (*C*_q bridge_), 27.7 (COOCH_2_*C*H_2_CH_2_), 21.4 (COOCH_2_CH_2_*C*H_2_), 13.8 (*C*H_3_).

^19^F{^1^H} NMR (471 MHz, DMSO-*d*_6_) *δ* (ppm) = −139.92 to −142.19 (m; *F*_*ortho*_), −156.21 to −158.23 (m; *F*_*meta*_).

IR (KBr) (*ν* cm^−1^): 3392 (OH), 2926 (C–H), 1740 (C=O).

### 3.4 Spectroscopic and photophysical characterization

The spectroscopic and photophysical characterization was performed in air-equilibrated dimethylsulfoxide (DMSO), dimethylformamide (DMF) or water using 0.5 cm path length quartz cells (Fig. S34 and S35). Fluorescence quantum yields were measured by the reference method using a solution of cresyl violet perchlorate in methanol as standard (*Φ*_F_ = 0.54).^45^ Several solutions were prepared in µM concentration range. To avoid non-linear effects, the absorbances were kept bellow 0.1 at the excitation wavelengths. The fluorescence spectra were measured for each sample and standard at the same conditions. The slopes (*m*) obtained from the linear fit of the integrated fluorescence intensity as a function of the absorbance at the excitation wavelength were used to calculate the emission quantum yield (*Φ*_F_) according to eqn (1):1
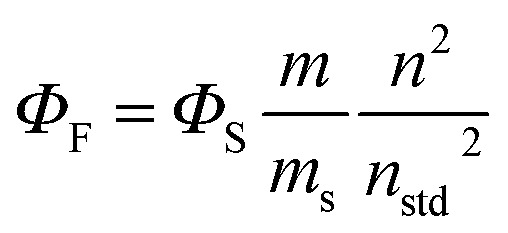
where the subscript std denoted the standard and *n* the refractive index of the solvent.

The singlet oxygen (^1^O_2_) generation efficiency of compounds 10–13 was evaluated in DMF using 9,10-dimethylanthracene (9,10-DMA) as a chemical probe for ^1^O_2_ detection.^52,53^ The absorbance of each PS sample was adjusted to 0.2 at 420 nm prior to irradiation. Experiments were carried out in quartz cuvettes, with irradiation provided by a Horiba Spex Fluoromax 4 Plus spectrofluorimeter equipped with an excitation source centered at 420 ± 5 nm. The oxidation of 9,10-DMA (∼50 µM) was monitored by tracking the decrease in absorbance at 378 nm every 120 s for a total of 600 s, using a UV-2501PC SHIMADZU spectrophotometer. For comparison, 5,10,15,20-tetraphenylporphyrin (TPP) was used as a reference standard due to its well-characterized photophysical properties.^53^ Control experiments confirmed that no significant photodegradation of 9,10-DMA occurred in the absence of a PS under identical irradiation conditions. All measurements were performed in triplicate to ensure reproducibility. ^1^O_2_ quantum yield (*Φ*_Δ_) was calculated using eqn (2).2
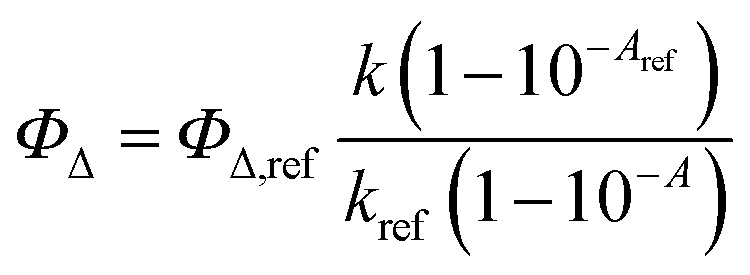
where the subscript ref refers to the TPP reference data, *k* and *A* are the photosensitized oxidation rate and absorbance at 420 nm, respectively.

The fluorescence decays were measured in 5 mm quartz cuvettes by the single-photon timing technique under excitation at 570 nm by collecting the emission at 670 nm (Fig. S36).

### 3.5 Biological assays

#### 3.5.1 Cell culture maintenance

Cervical carcinoma (HeLa) cell line, used as cancer cell models, was purchased from ECACC (European Collection of Authenticated Cell Cultures). Cells were cultured in Dulbecco's Modified Eagle Medium, DMEM (GIBCO™), supplemented with 10% fetal bovine serum (FBS, GIBCO™) and 1% penicillin/streptomycin (PS, GIBCO™) and maintained in a humidified atmosphere with 5% CO_2_ at 37 °C. The subcultures were maintained routinely using TrypLE Express without phenol red, GIBCO™, for chemical detaching. Cells were counted with a Neubauer chamber.

#### 3.5.2 Assessment of cellular viability and phototoxicity

The cytotoxicity of compounds 10, 11, 12, and 13 in the absence of light was initially assessed in HeLa cells. Cells were seeded in flat-bottom 96-well polystyrene plates at a density of 1 × 10^4^ cells per well and incubated overnight at 37 °C in a humidified atmosphere containing 5% CO_2_ to allow for cell adhesion according to IUPAC. After 24 h, the culture medium was replaced with fresh medium containing increasing concentrations (0, 0.625, 1.25, 2.5, 5, and 10 µM) of 10 and 11 in 0.1% of DMSO and 12 and 13 in 10% of H_2_O. Cells were then incubated for 12 h, after which viability was measured for non-irradiated conditions.

Phototoxicity was evaluated following the same cell seeding and compound treatment protocol. After the 12 h pre-incubation with the compounds, cells were exposed to blue light (*λ* = 420 nm) using a Unilight irradiation system at energy doses of 1, 5, and 10 J cm^−2^. Cell viability was assessed 24 h post-irradiation.

Viability was determined using the PrestoBlue™ reagent (Invitrogen, Carlsbad, CA, USA), following the manufacturer's protocol. Fluorescence intensity was measured at 590 nm using a PolarStar Optima microplate reader (BMG Labtech). The percentage of metabolically active cells was calculated based on the reduction of resazurin and expressed relative to untreated control cells, with normalization to the negative control. For phototoxicity assays, irradiated samples were normalized to their respective dark (non-irradiated) controls. All experiments were performed in triplicate.

#### 3.5.3 Statistical analysis

IC_50_ (µM) values were determined for compounds 10–13 using GraphPad Prism software. Data were analysed by nonlinear regression with the log(inhibitor) *vs.* response–variable slope (four parameters) model.

## 4. Conclusions

In this study, new alkyne-substituted Ga(iii) corrole complexes were prepared from Ga(iii) complex of 5,10,15-tris(pentafluorophenyl)corrole and propargylic alcohol *via* nucleophilic aromatic substitution under controlled conditions. These complexes were subsequently conjugated to an azide functionalized glycofullerene *via* copper-catalyzed azide alkyne cycloaddition reactions. The successful synthesis of the target nanostructures, was confirmed by a combination of standard spectroscopic techniques, including ^1^H NMR, ^13^C NMR, HRMS, FTIR, and UV-Vis spectroscopy, with the glycofullerene corrole conjugates obtained in appreciable yields exceeding 70%. All compounds exhibited the characteristic optical features of corrole, including a strong Soret band near 425 nm and several weaker Q bands between 500 and 660 nm. The UV-Vis absorption spectra of the conjugates were dominated by the corrole unit, with no significant spectral shifts observed upon conjugation to the glycofullerene, indicating minimal ground-state electronic interaction between the two moieties. Similarly, fluorescence measurements showed that both the precursor corroles and the mono- and tris-functionalized conjugates emitted around 610 nm, consistent with typical corrole fluorescence. The glycofullerene moiety did not contribute to the emission, in line with its non-emissive nature. These results confirm that the photophysical properties of the conjugates are primarily dominated by the corrole core. The PDT potential of compounds was evaluated through *in vitro* assays using HeLa cells. The HeLa cells were irradiated at 420 nm with varying light doses (1, 5, and 10 J cm^−2^) to assess the phototoxicity of the compounds under controlled light exposure. Among the tested compounds, the mono- and tris-alkyne-substituted corroles as well as the mono glycofullerene–corrole conjugate showed the most promising photosensitizing activity, with IC_50_ values below 1 µM at a light dose as low as 5 J cm^−2^. Importantly, none of those compounds exhibited observable dark toxicity at concentrations up to 20 µM, which was the highest concentration tested.

Taken together, these results demonstrate the promise of glycofullerene hybrids bearing a corrole unit as efficient, water-soluble, and stable photosensitizers, paving the way for next-generation nanotherapeutics in cancer treatment. Future work should focus on synthesizing individual regioisomers in which only one C_6_F_5_ substituent serves as the coupling site to the fullerene, while the remaining meso positions bear distinct aryl groups. The potential role of the glycofullerene moiety in mediating interactions with lectins or sugar transporters could also be investigated to explore opportunities for enhanced cellular uptake or targeting of cancer cells. Such studies will clarify structure–activity relationships and further optimize photodynamic performance.

## Author contributions

Jennifer Patino-Alonso: synthesis, photophysical studies, data analysis, writing – original draft. Carla I. M. Santos: synthesis, photophysical studies, data analysis, writing – original draft, writing – review & editing. Adriana F. Cruz: biological assays. Sandra Pinto: biological assays. Justo Cabrera-González: formal analysis, review and validation. M. Amparo F. Faustino: validation, analysis and review. M. Graça P. M. S. Neves: validation, data analysis and review. Ermelinda M. S. Maçôas: confocal microscopy, analysis, validation and review. Nazario Martín: design, formal analysis, supervision, funding acquisition. Beatriz M. Illescas: design, methodology, formal analysis, writing, review, supervision, funding acquisition.

## Conflicts of interest

There are no conflicts to declare.

## Supplementary Material

SC-OLF-D5SC06977G-s001

## Data Availability

All the data supporting this article are available in the supplementary information (SI). Additional datasets or information are available from the corresponding author upon reasonable request. Supplementary information is available. See DOI: https://doi.org/10.1039/d5sc06977g.
