# Supplementary material for: Glycofullerene–corrole hybrids: a new class of multifunctional nanomaterials with potential in targeted photodynamic therapy
Source: Chem Sci. 2025 Dec 8;17(6):3339–50. doi: 10.1039/d5sc06977g (PMC12721340; doi:10.1039/d5sc06977g)
Supplement: SC-017-D5SC06977G-s001 [file SC-017-D5SC06977G-s001.pdf]

Supplementary Information (SI) for Chemical Science.
This journal is © The Royal Society of Chemistry 2025

Supporting Information

Glycofullerene–Corrole Hybrids: A new class of multifunctional nanomaterials with potential in targeted photodynamic therapy

Jennifer Patino-Alonso,[a, b, c] Carla I. M. Santos,*[b,c] Adriana F. Cruz,[d] Sandra Pinto,[d] Justo Cabrera-González,[a, e] M. Amparo F. Faustino,[c] M. Graça P. M. S. Neves,[c] Ermelinda M. S. Maçôas,*[b] Nazario Martín,*[a,f] Beatriz M. Illescas*[a]

[a] Departamento de Química Orgánica, Facultad de Química, Universidad Complutense, E-28040 Madrid, Spain.

[b] Centro de Química Estrutural, Institute of Molecular Sciences, Departamento de Engenharia Química, Instituto Superior Técnico, Universidade de Lisboa, 1049-001 Lisboa, Portugal.

[c] LAQV-REQUIMTE and Department of Chemistry, University of Aveiro, Campus Universitário de Santiago, 3810-193 Aveiro, Portugal.

[d] iBB- Institute for Bioengineering and Biosciences, Instituto Superior Técnico, Av. Rovisco Pais, 1049-001 Lisboa, Portugal.

[e] Departamento de Química en Ciencias Farmacéuticas, Facultad de Farmacia, Universidad Complutense, E-28040 Madrid, Spain.

[f] IMDEA-Nanoscience, C/ Faraday 9, Campus de Cantoblanco, 28049 Madrid, Spain.

Supplementary Information (SI) contains: Additional information on biological assays, synthesis, structural and photophysical characterization (Schemes S1-S2, Figures S1-S37).

1. Synthesis and characterization

1.1. Synthetic pathways and structural characterization (^{1}H-NMR, ^{19}F-NMR, ^{13}C-NMR, FTIR, MS)

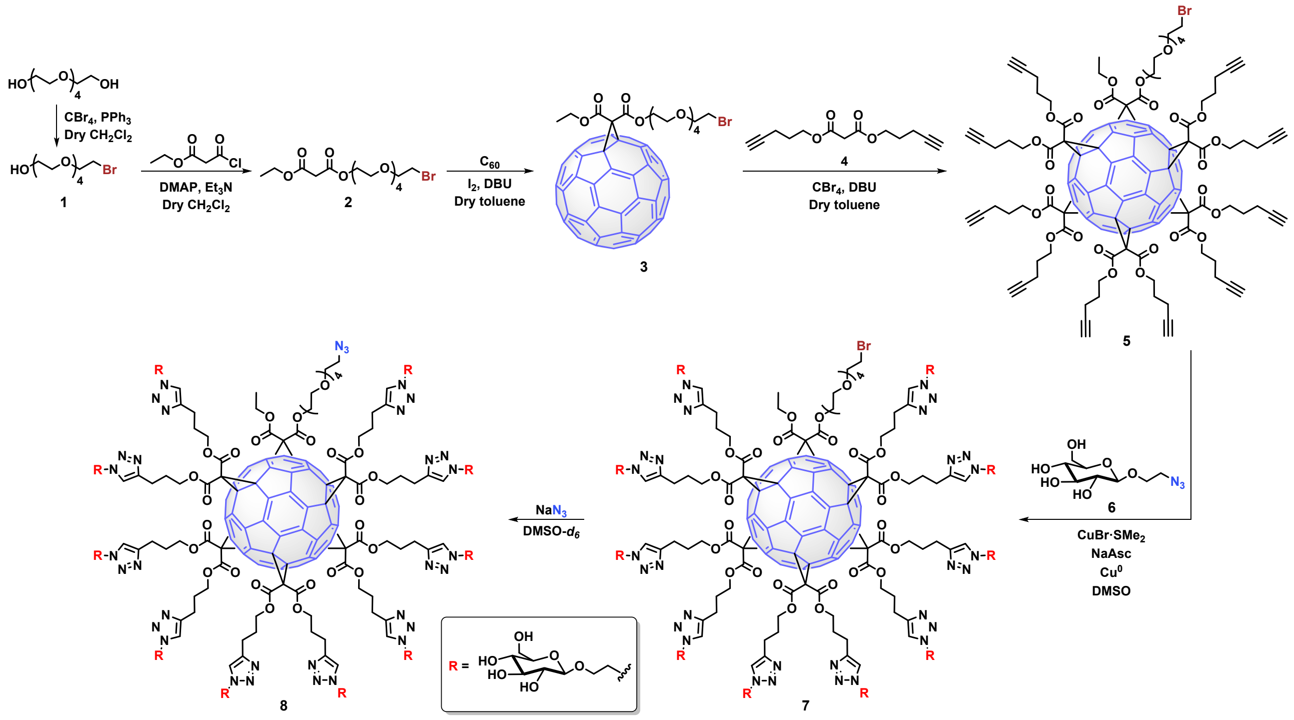

Scheme S1. *Synthetic pathway to obtain azide-substituted glycofullerene **8**.*

Compound 1: 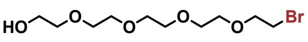

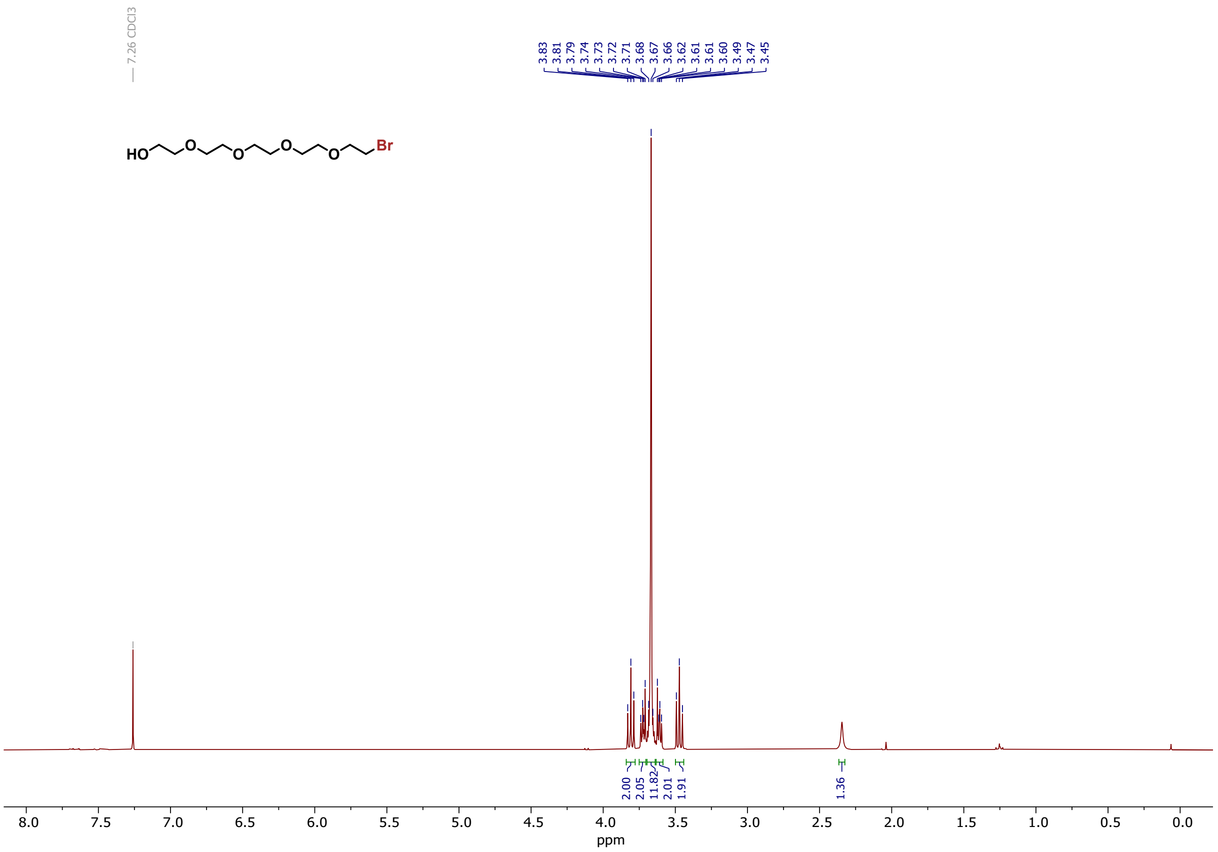

Figure S1. *^{1}H NMR spectrum of compound **1** in $CDCl_3$.*

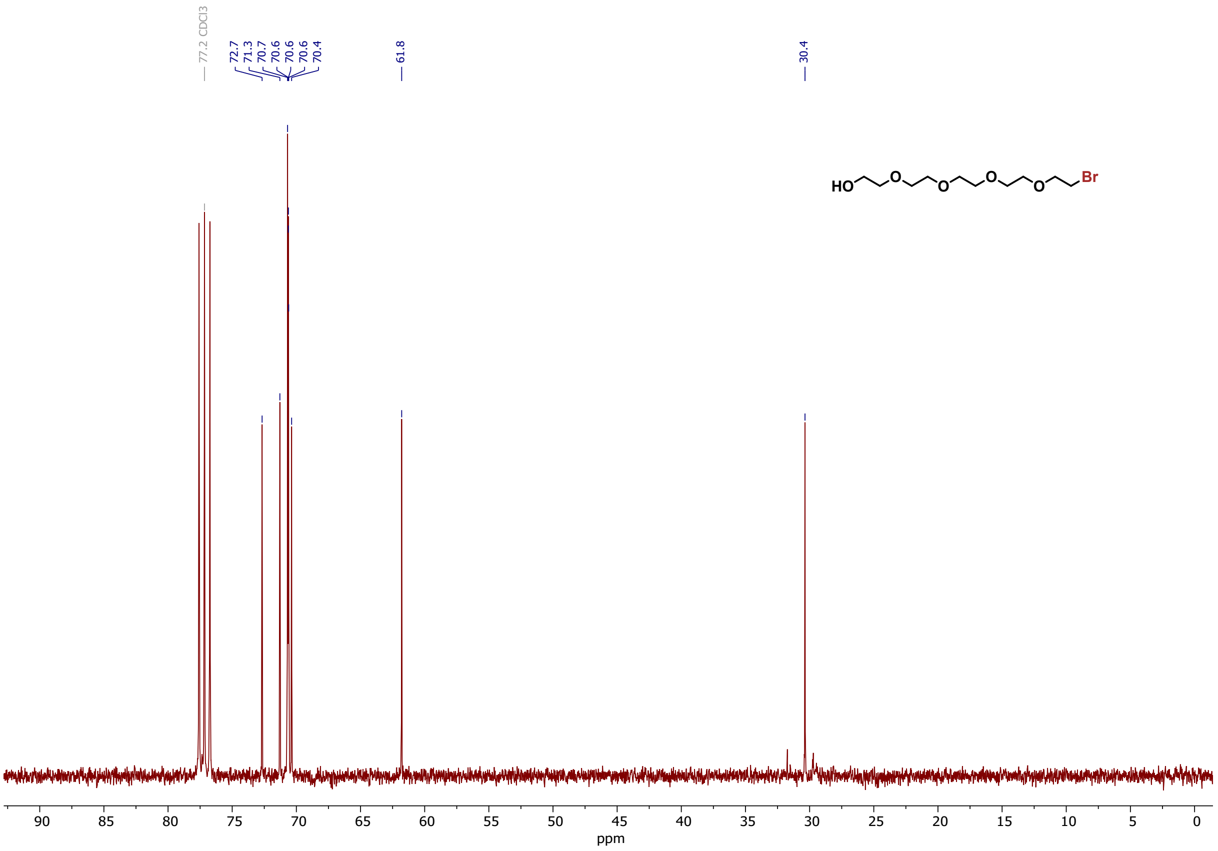

Figure S2. *^{13}C NMR spectrum of compound **1** in $CDCl_3$.*

Compound 2: 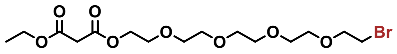

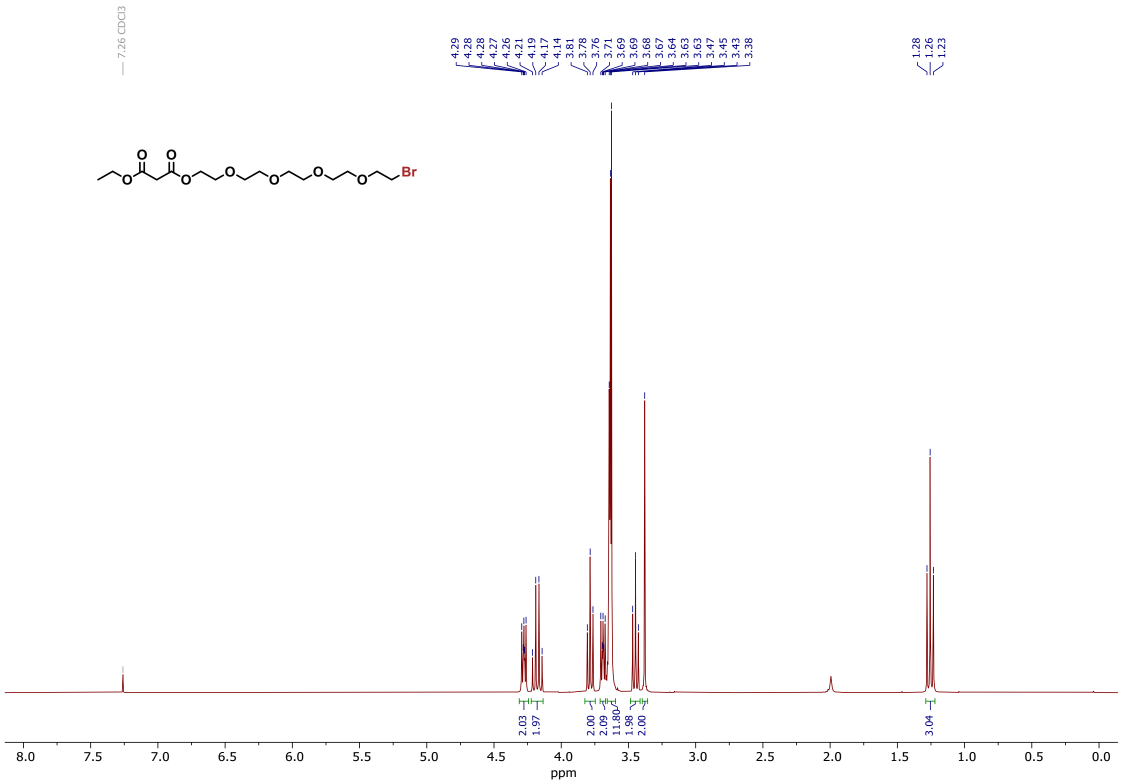

Figure S3. ^{1}H NMR spectrum of compound **2** in $CDCl_3$.

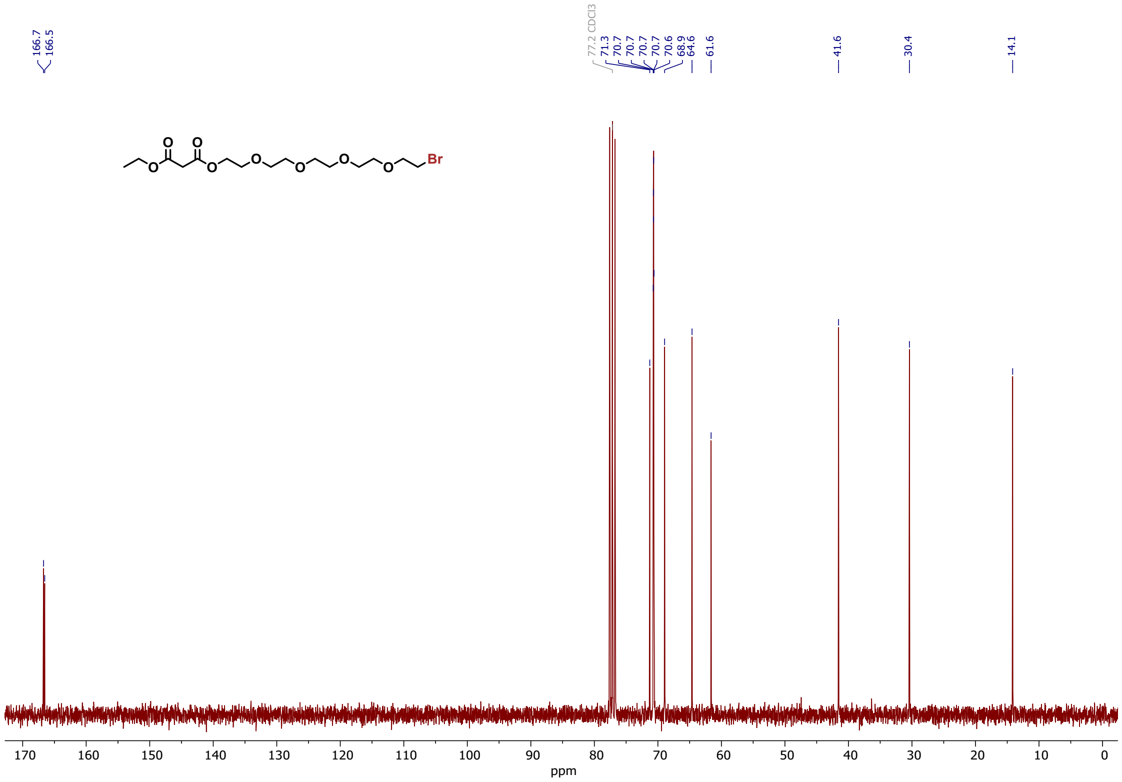

Figure S4. ^{13}C NMR spectrum of compound **2** in $CDCl_3$.

Compound 5:

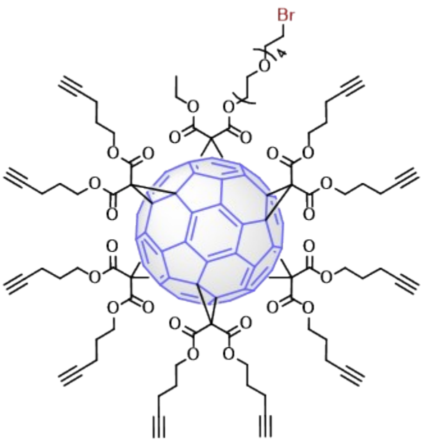

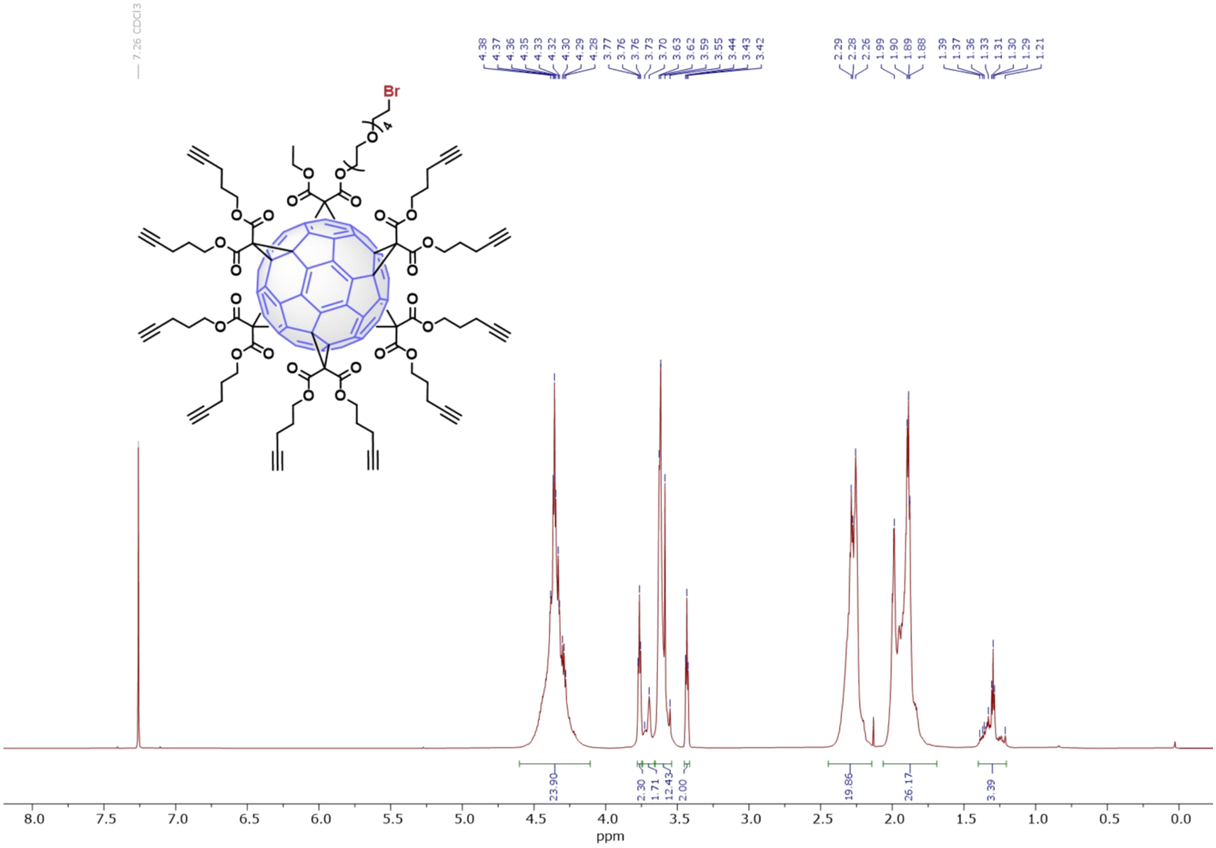

Figure S5. *^{1}H NMR spectrum of compound **5** in $CDCl_3$.*

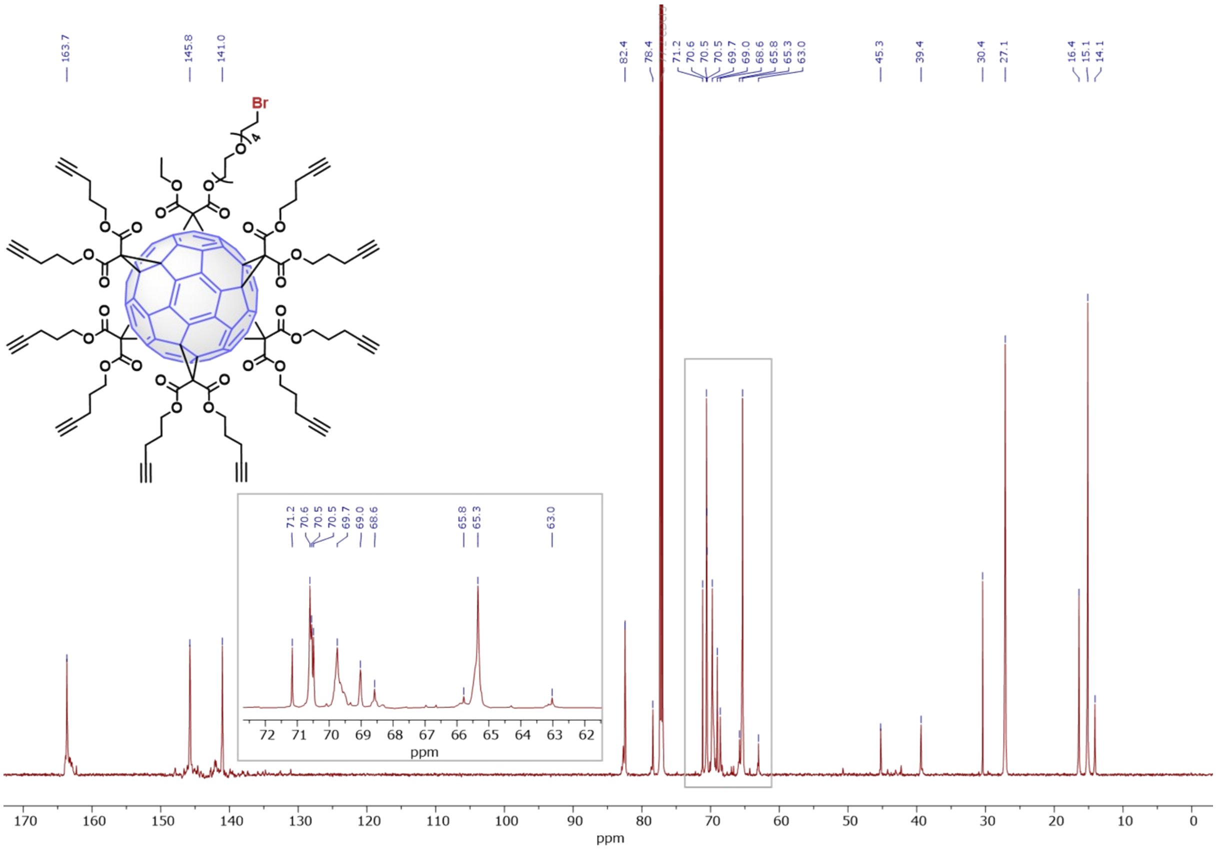

Figure S6. *^{13}C NMR spectrum of compound **5** in $CDCl_3$.*

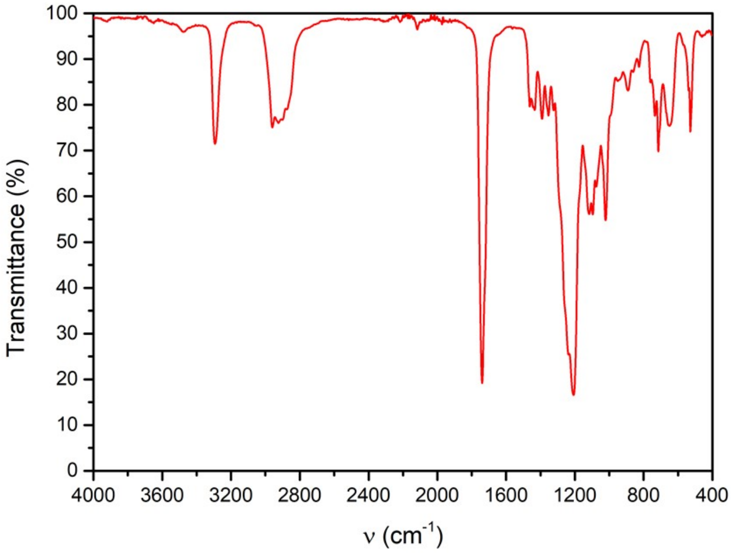

Figure S7. *FTIR-ATR spectrum of compound **5**.*

Compound 7:

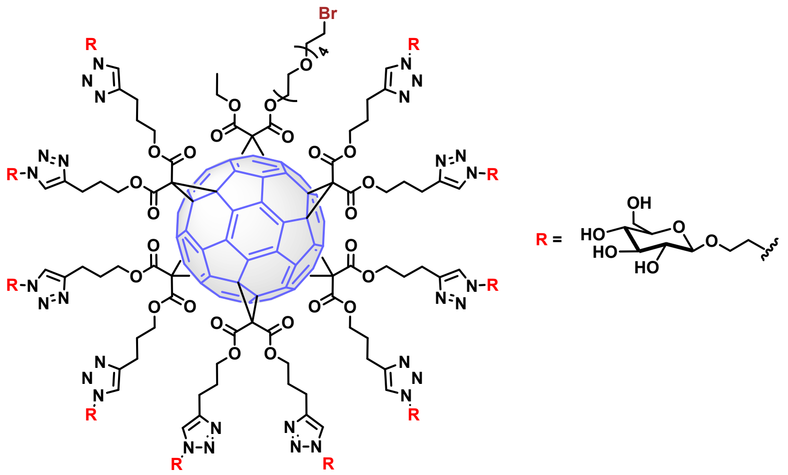

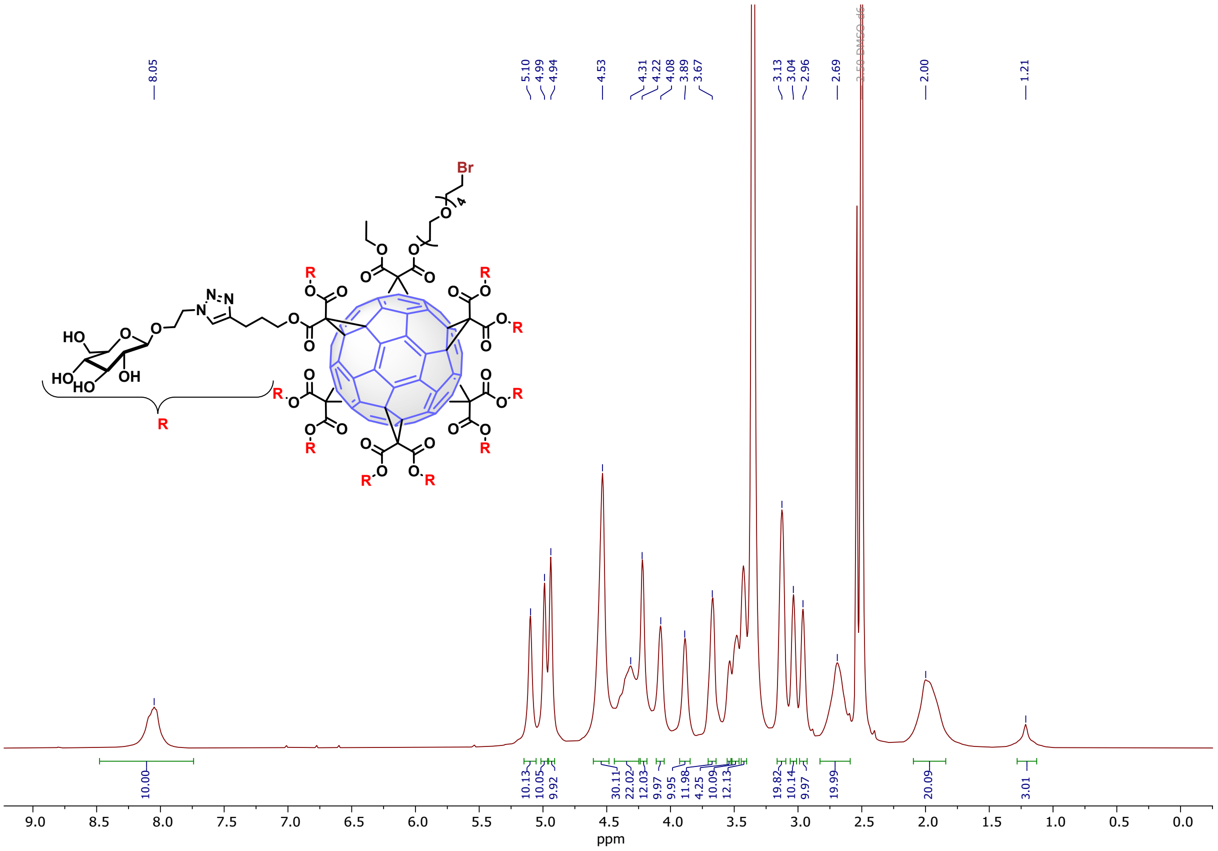

Figure S8. *^{1}H NMR spectrum of compound **7** in DMSO-d_6.*

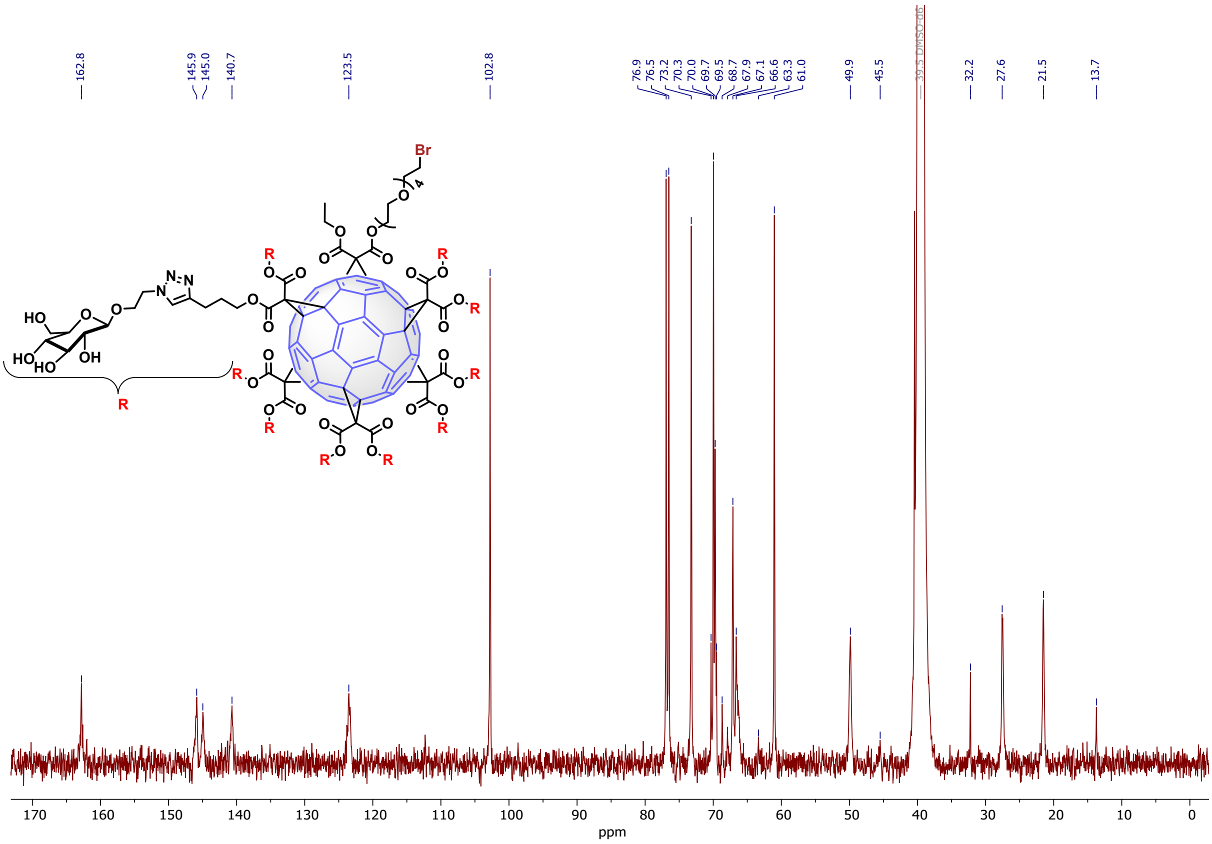

Figure S9. *^{13}C NMR spectrum of compound **7** in DMSO-d$_6$.*

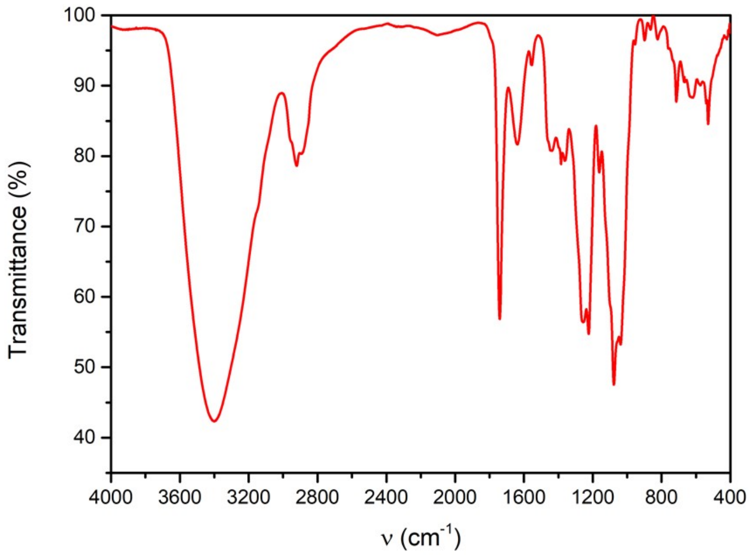

Figure S10. *IR (KBr) spectrum of compound **7**.*

Compound 8:

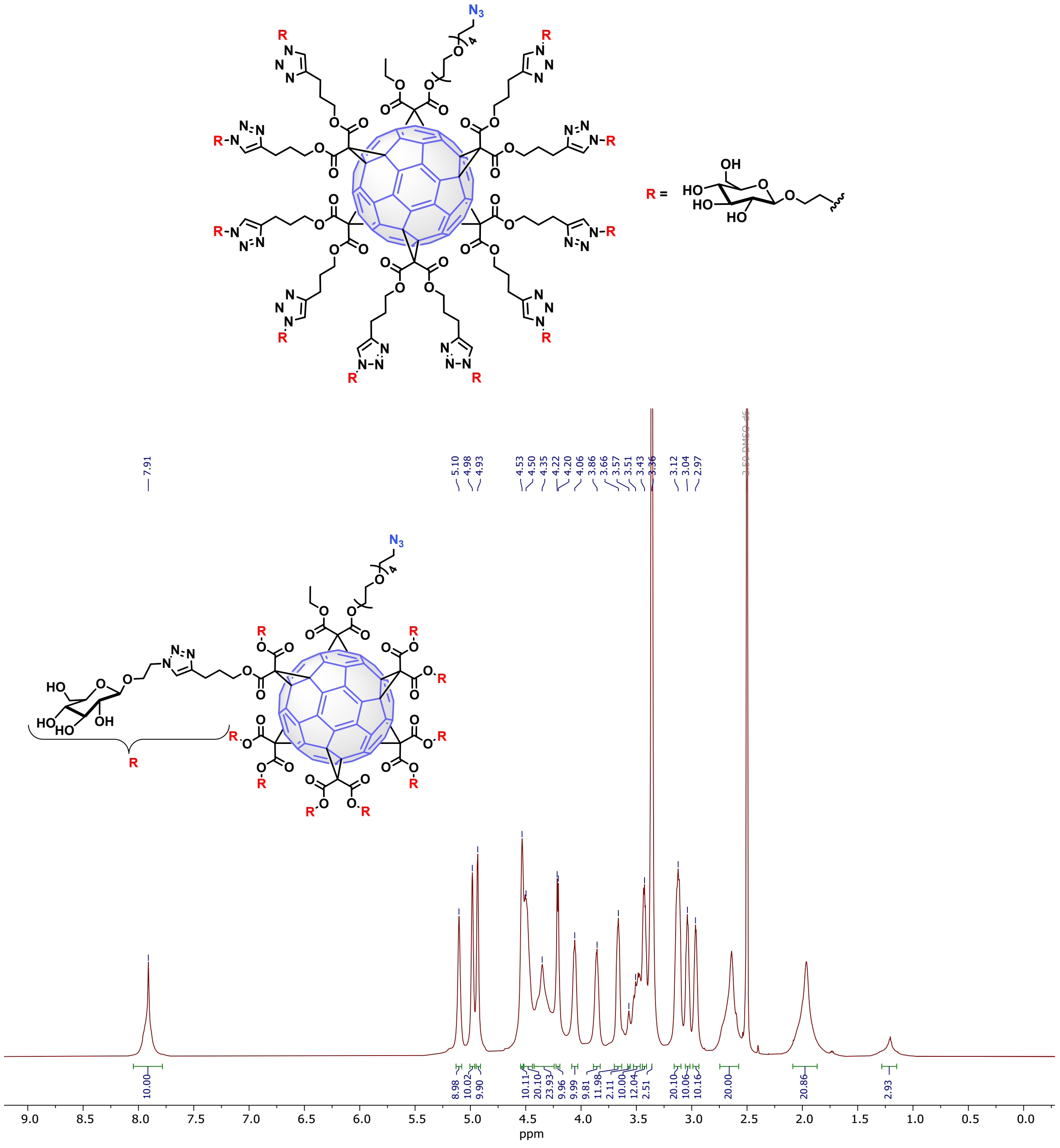

Figure S11. *^1H NMR spectrum of compound **8** in DMSO-d_6.*

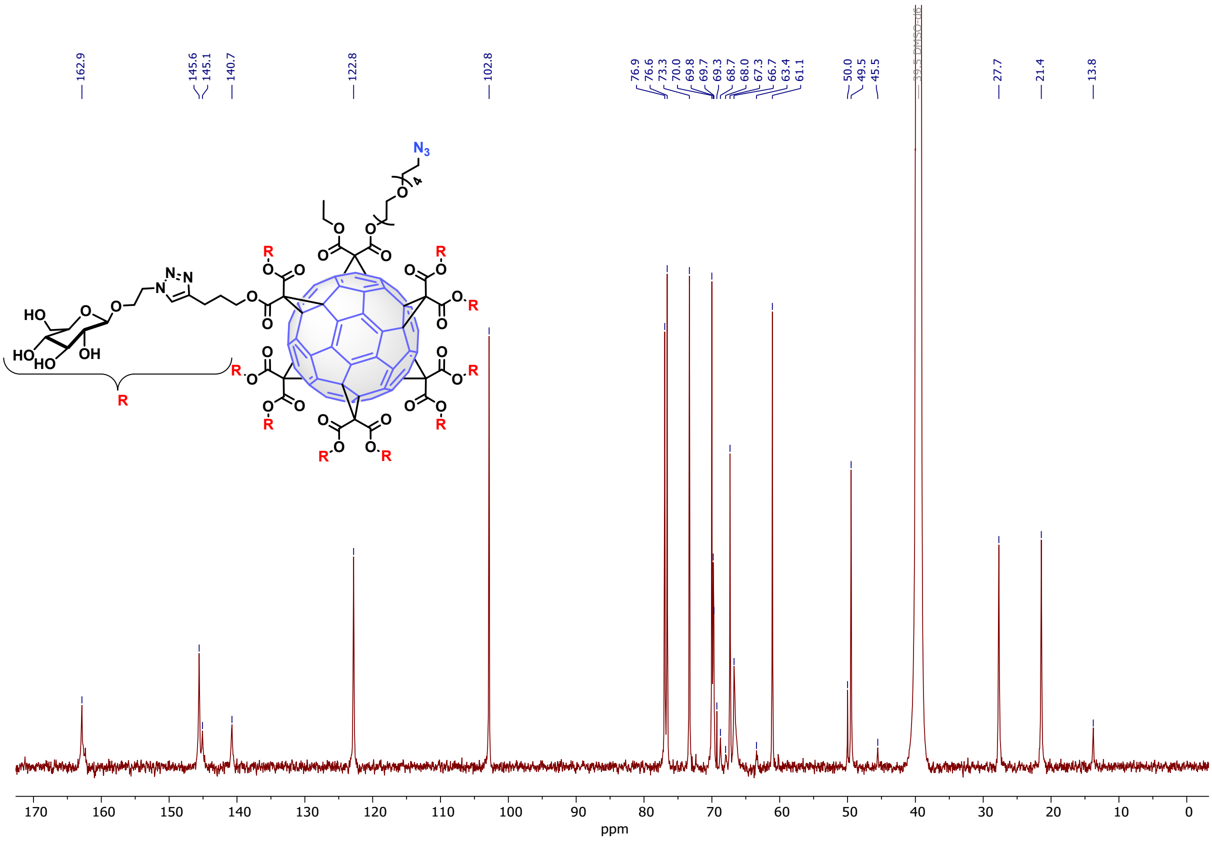

Figure S12. *^{13}C NMR spectrum of compound **8** in DMSO-d$_6$.*

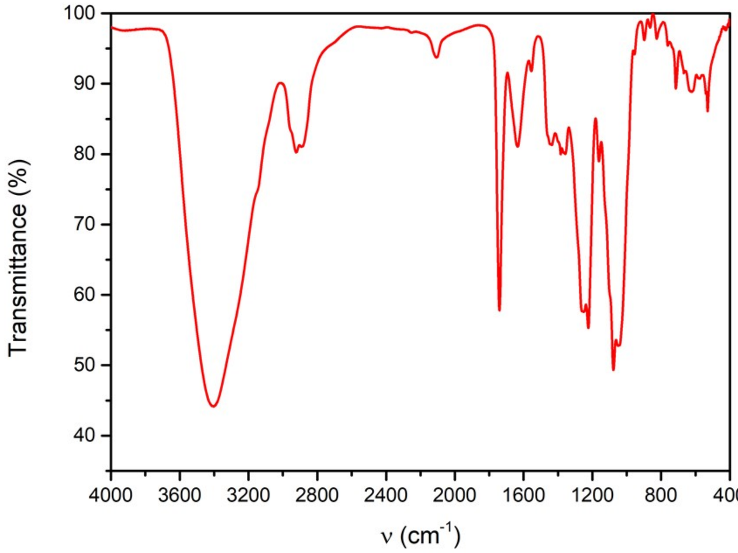

Figure S13. *IR (KBr) spectrum of compound **8**.*

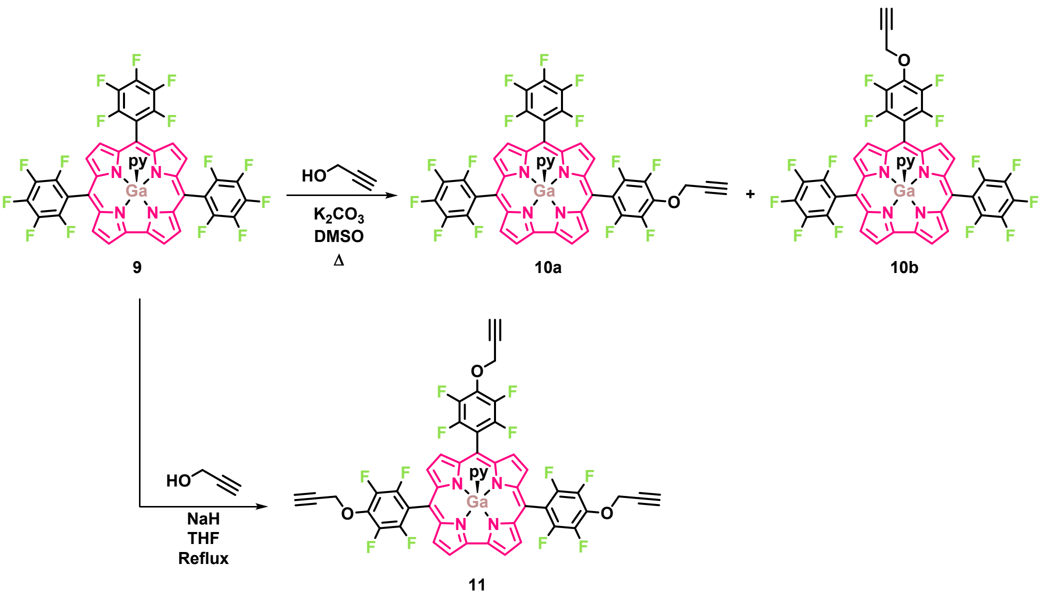

Scheme S2. *Synthetic pathway to obtain corroles* ***10*** *and* ***11****.*

Compound 10:

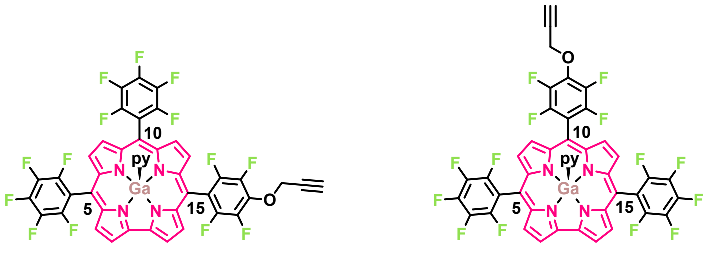

10a **10b**

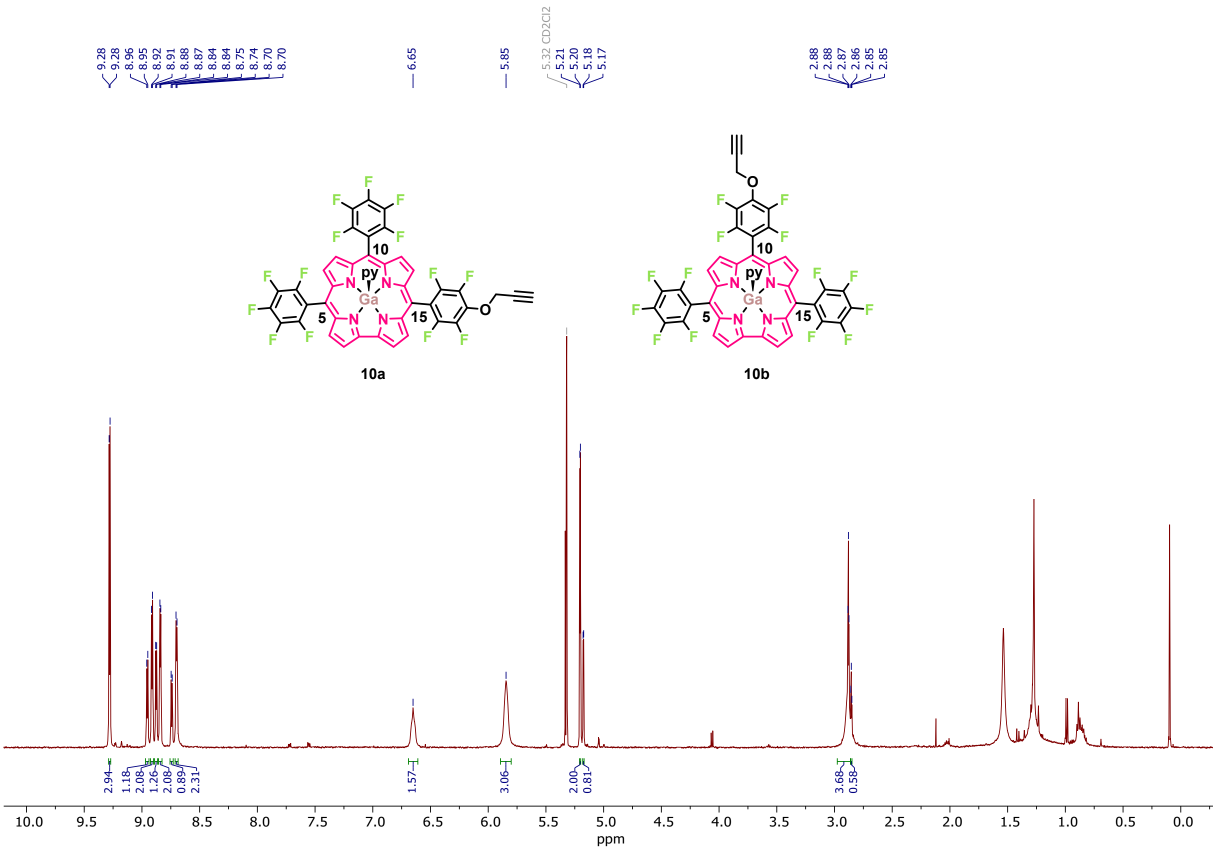

Figure S14. *^{1}H NMR spectrum of compound **10** in CD_2Cl_2.*

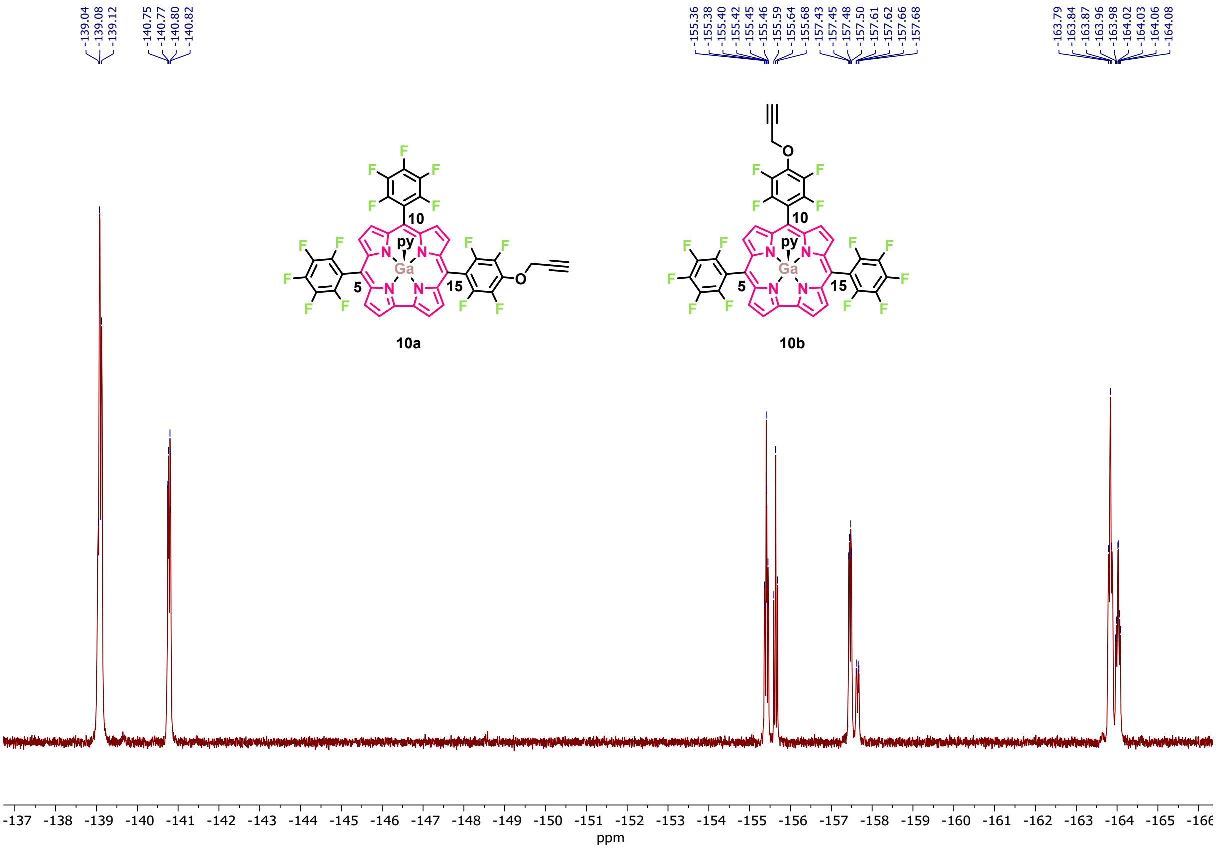

Figure S15. *^{19}F NMR spectrum of compound **10** in CD_2Cl_2.*

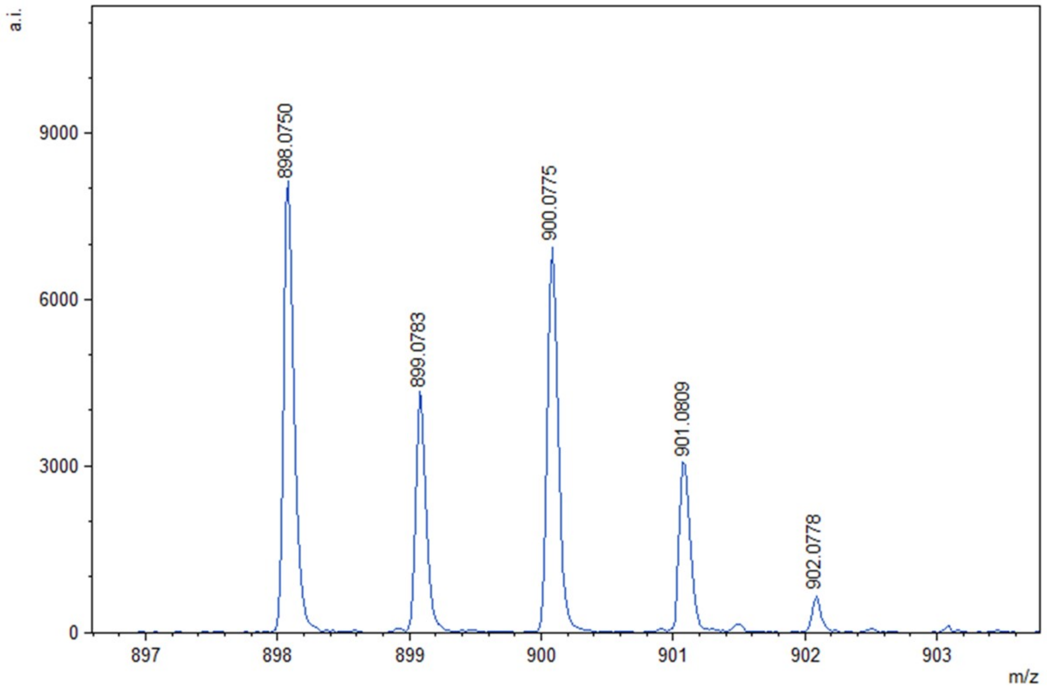

Figure S16. *MS (MALDI-TOF) of compound **10**.*

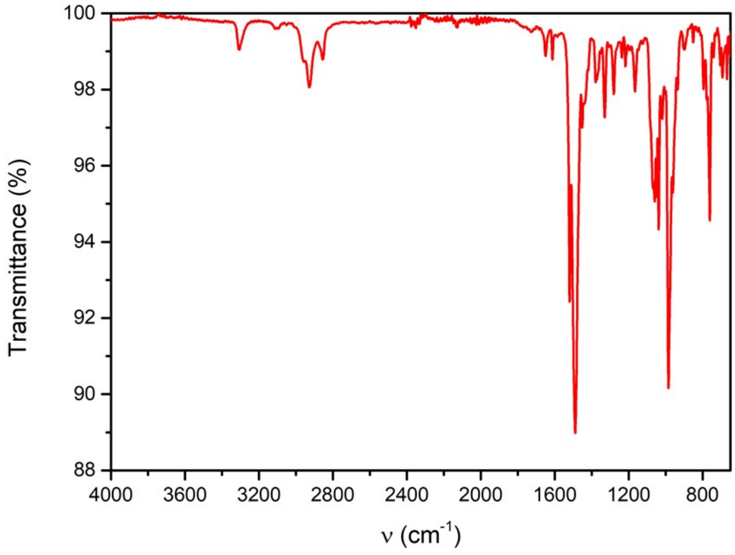

Figure S17. *FTIR-ATR spectrum of compound **10**.*

Compound 11:

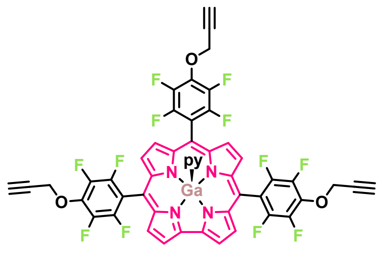

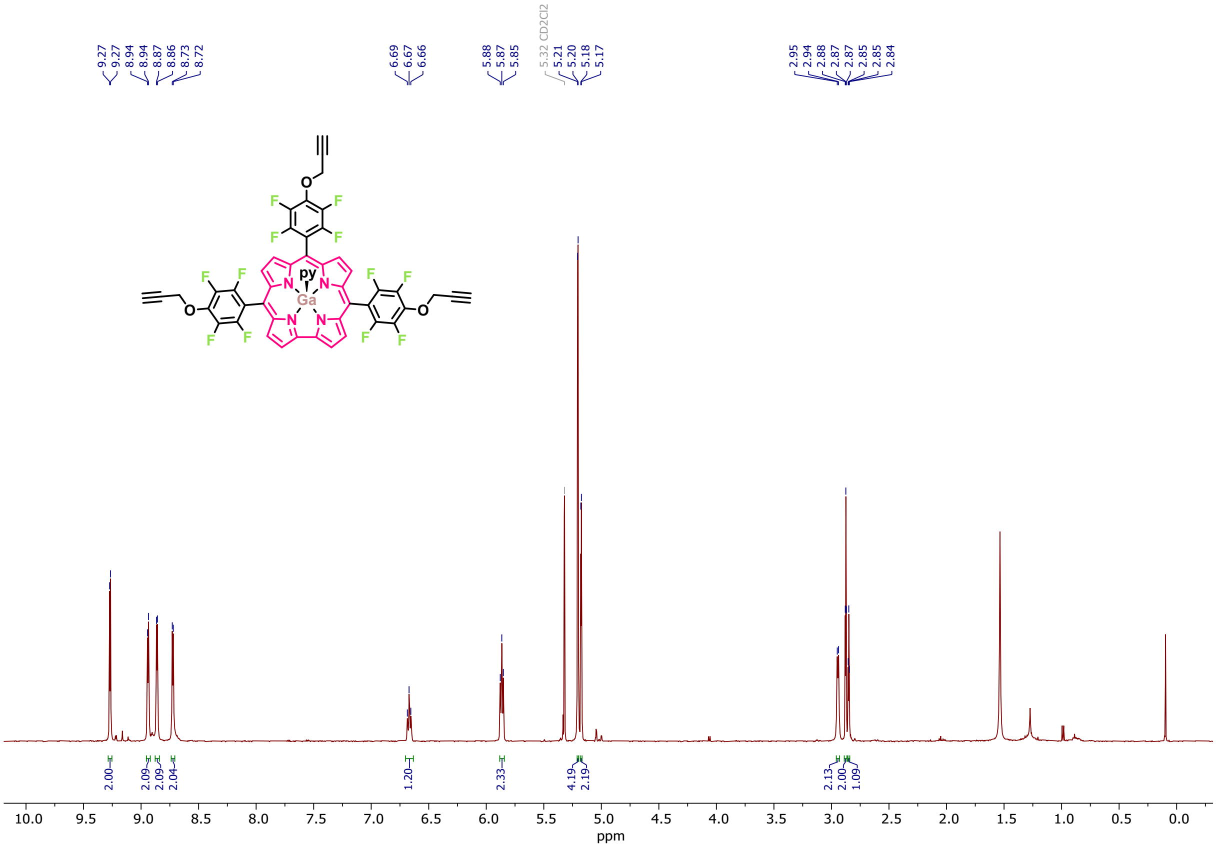

Figure S18. *^{1}H NMR spectrum of compound **11** in CD_2Cl_2.*

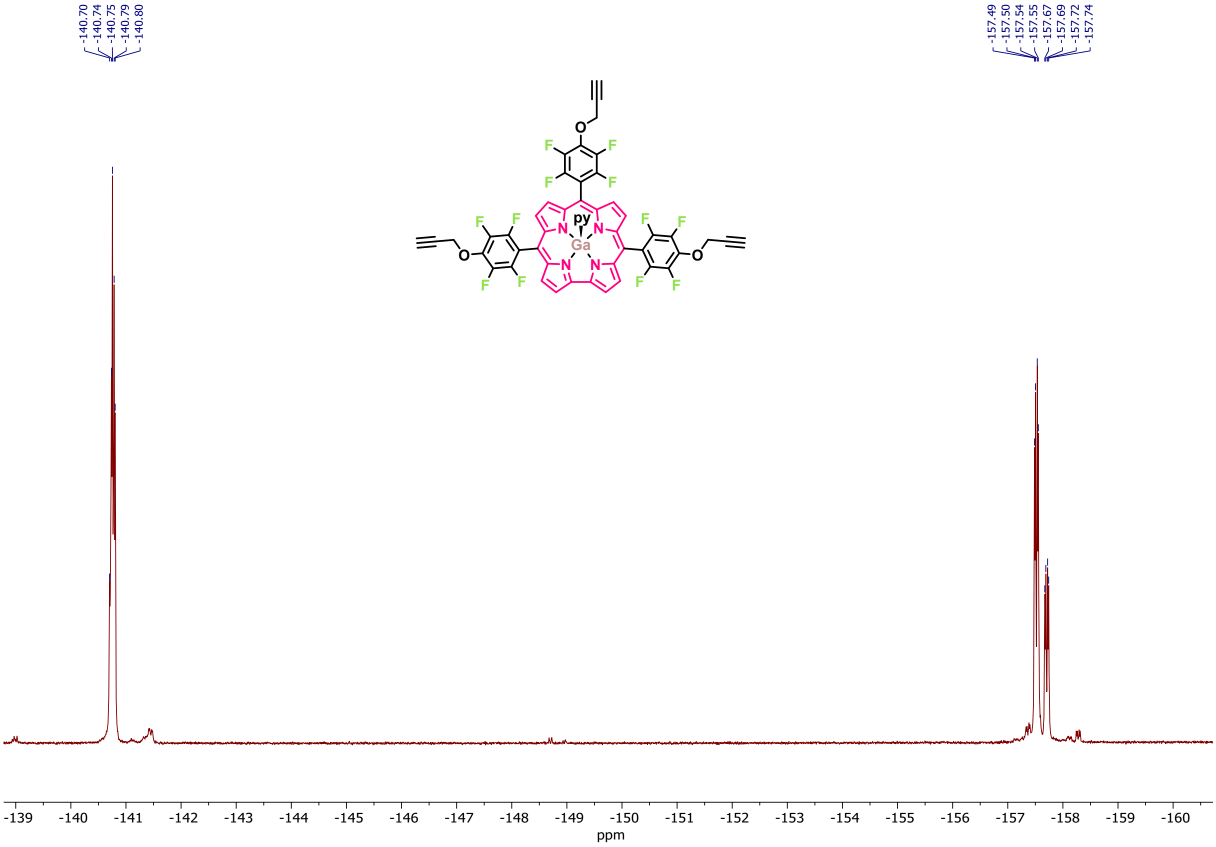

Figure S19. *^{19}F NMR spectrum of compound **11** in CD_2Cl_2.*

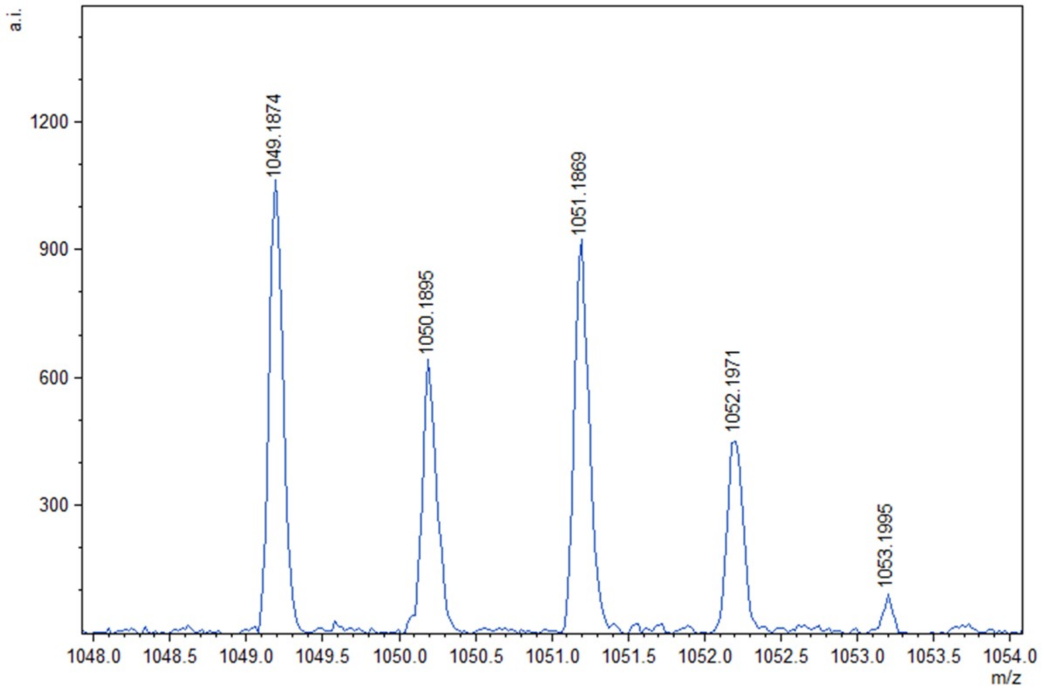

Figure S20. *MS (MALDI-TOF) of compound **11**.*

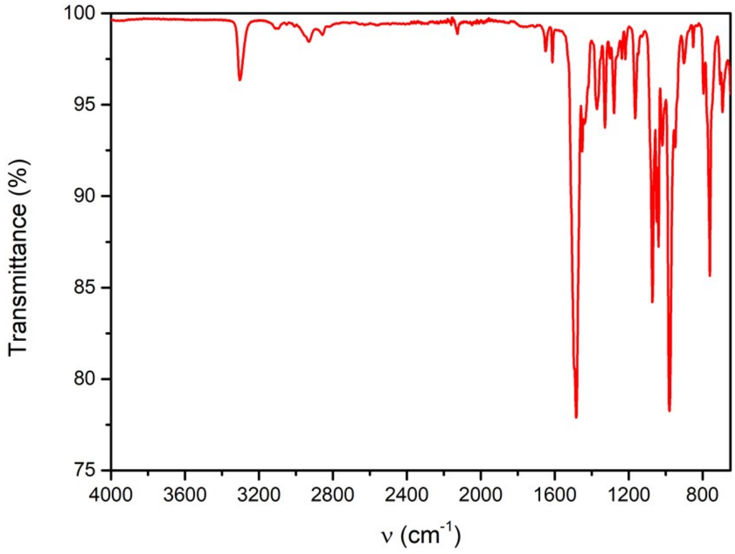

Figure S21. *FTIR-ATR spectrum of compound* **11**.

Compound 12:

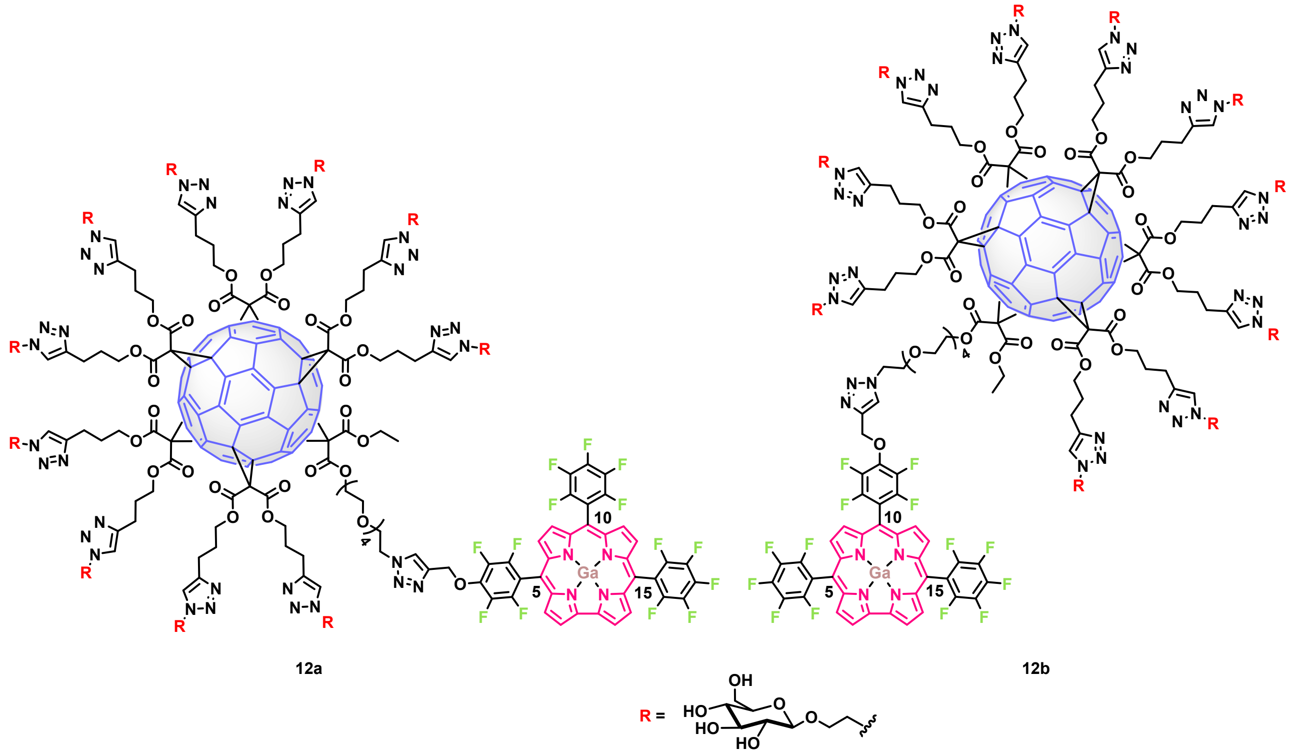

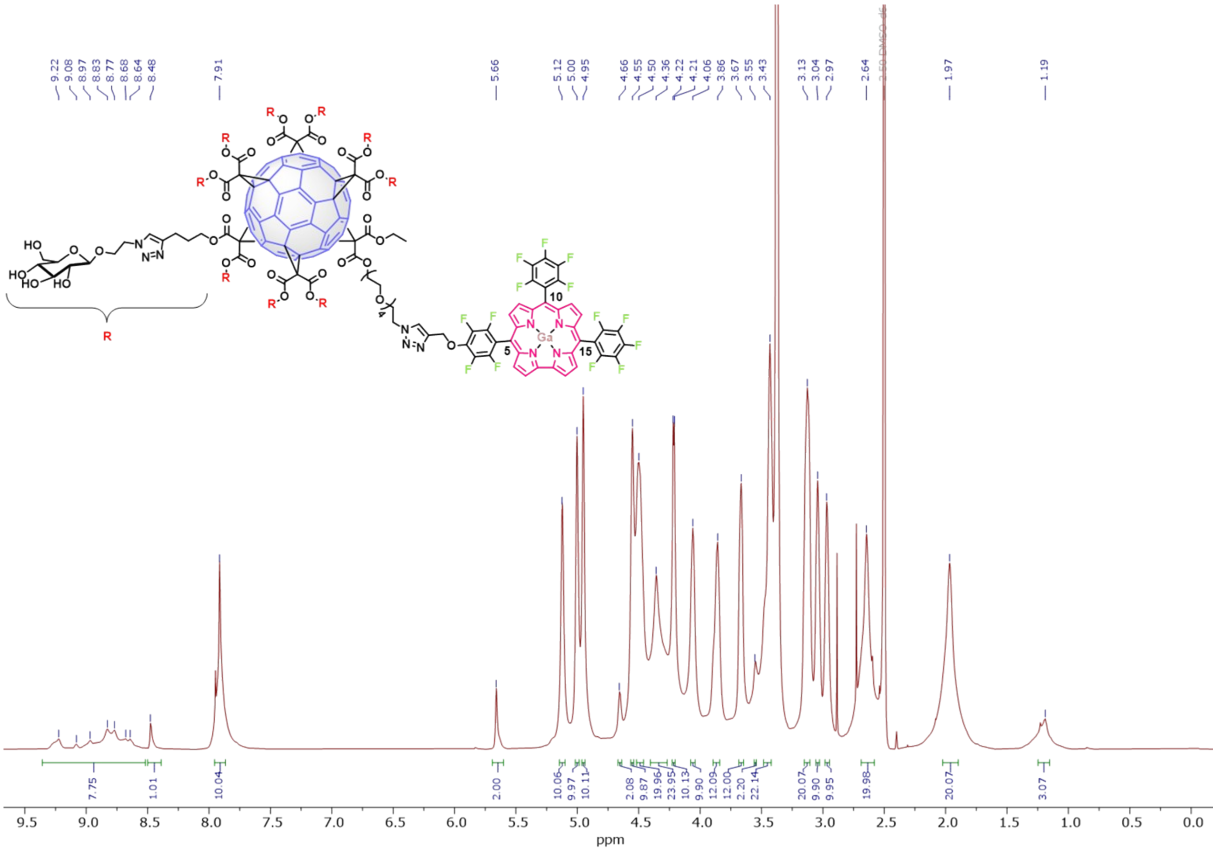

Figure S22. *^{1}H NMR spectrum of compound **12** in DMSO-d$_6$.*

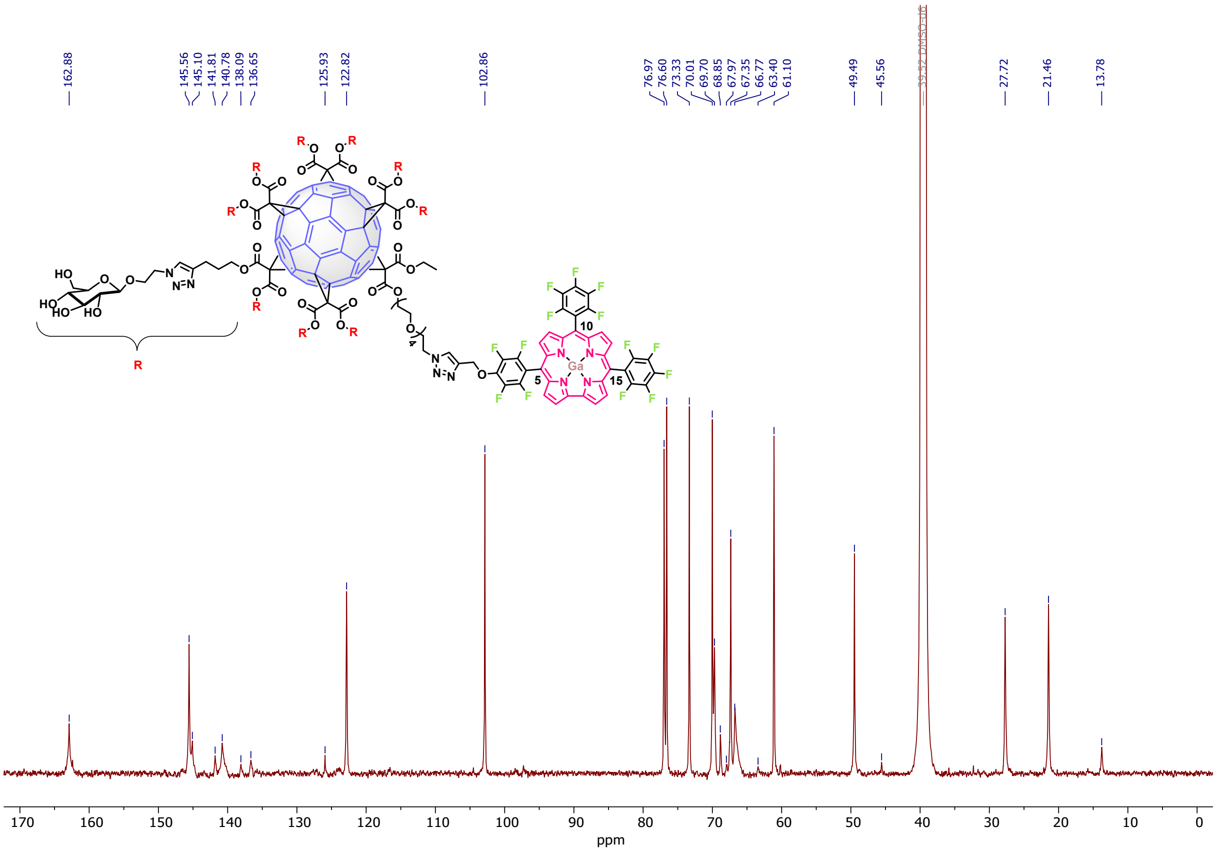

Figure S23. *^{13}C NMR spectrum of compound* ***12*** *in DMSO-d$_6$.*

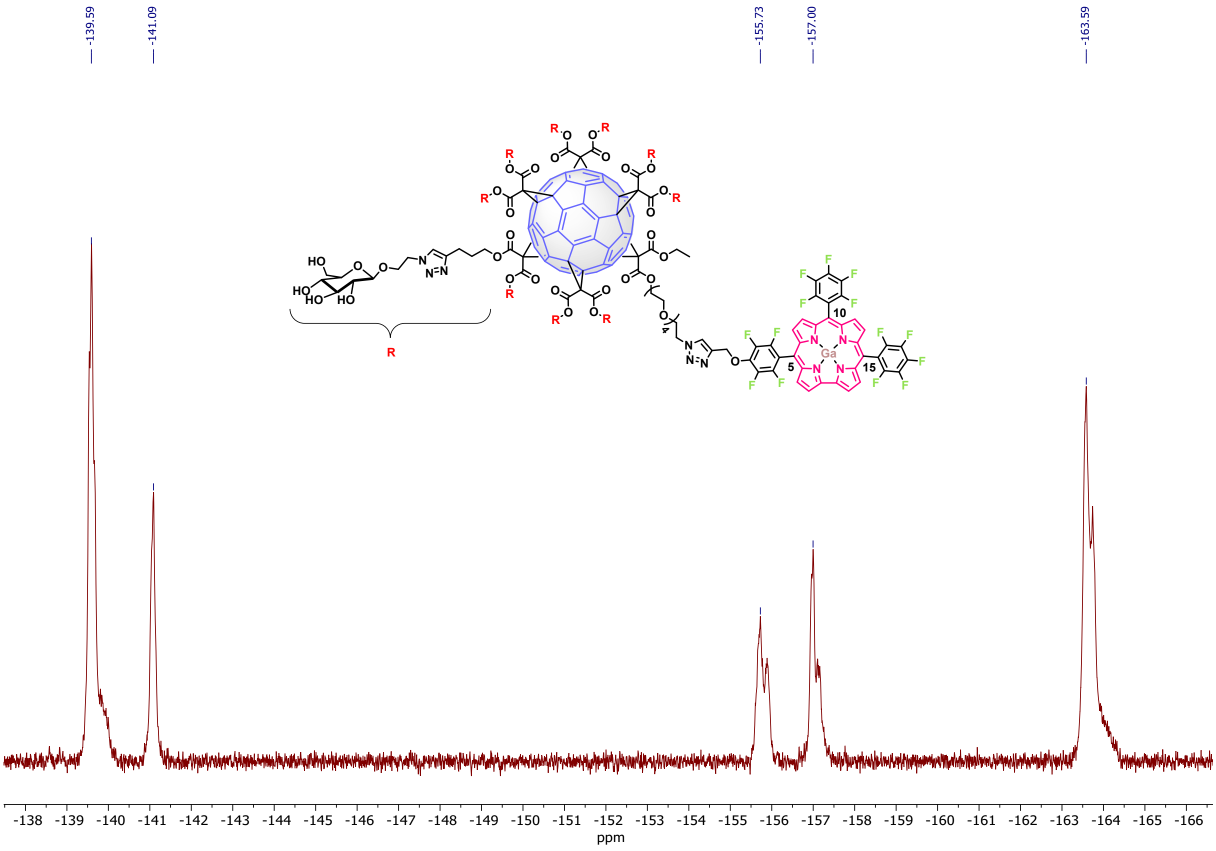

Figure S24. *^{19}F NMR spectrum of compound* ***12*** *in DMSO-d$_6$.*

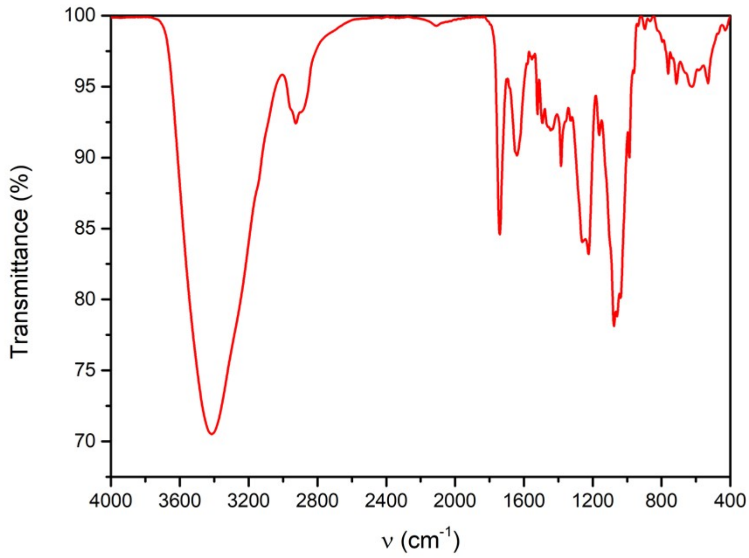

Figure S25. *IR (KBr) spectrum of compound* ***12****.*

Compound 13:

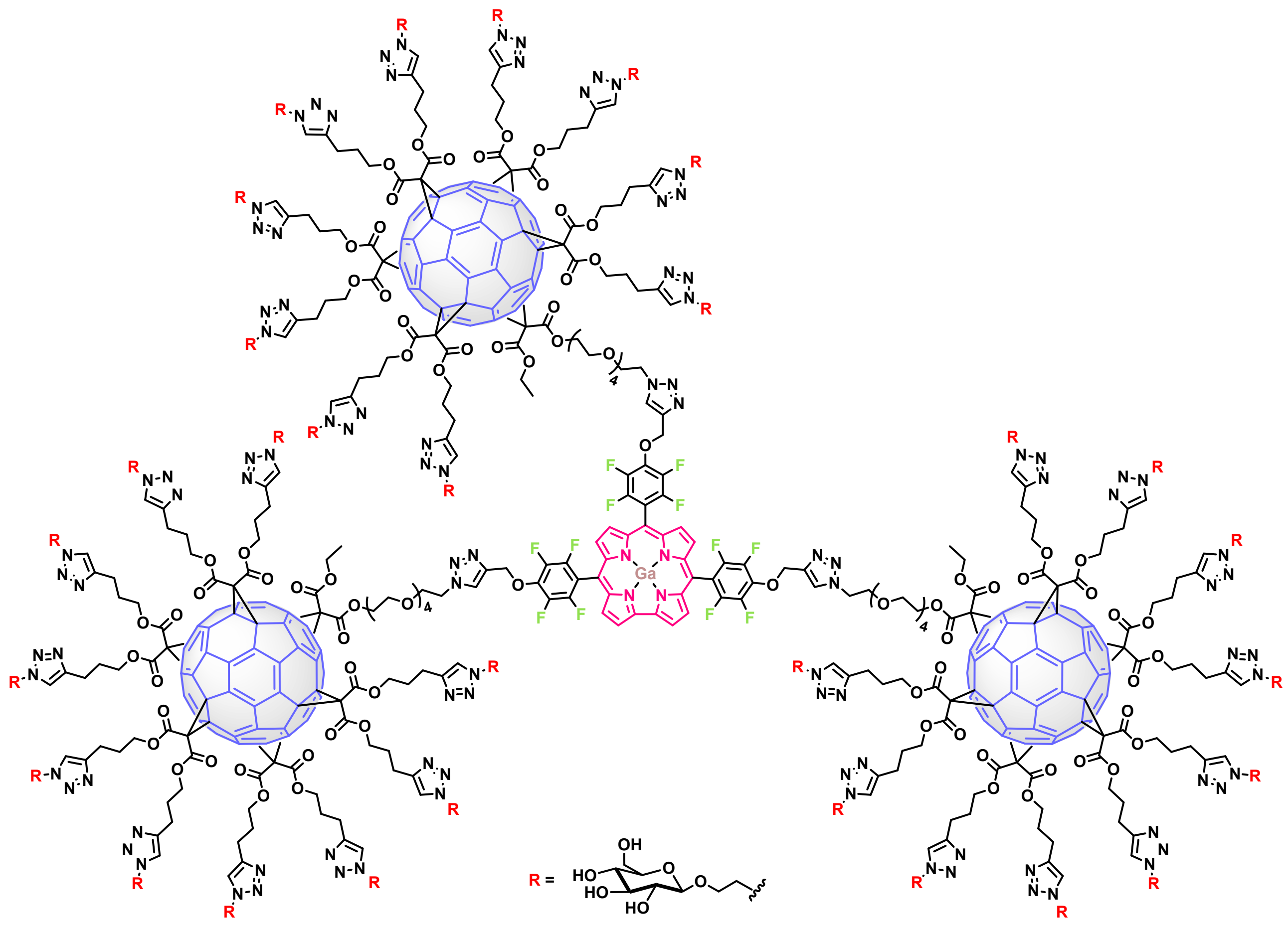

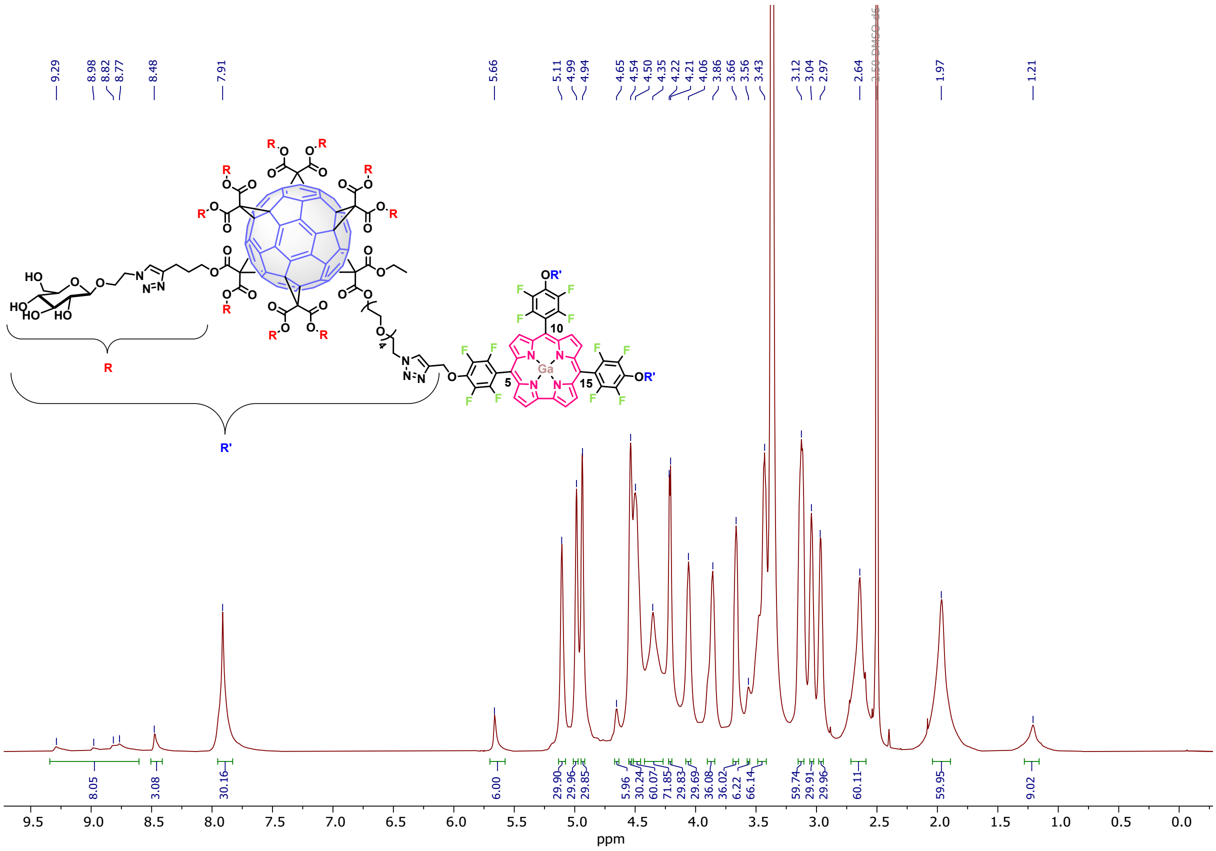

Figure S26. *^{1}H NMR spectrum of compound **13** in DMSO-d_6.*

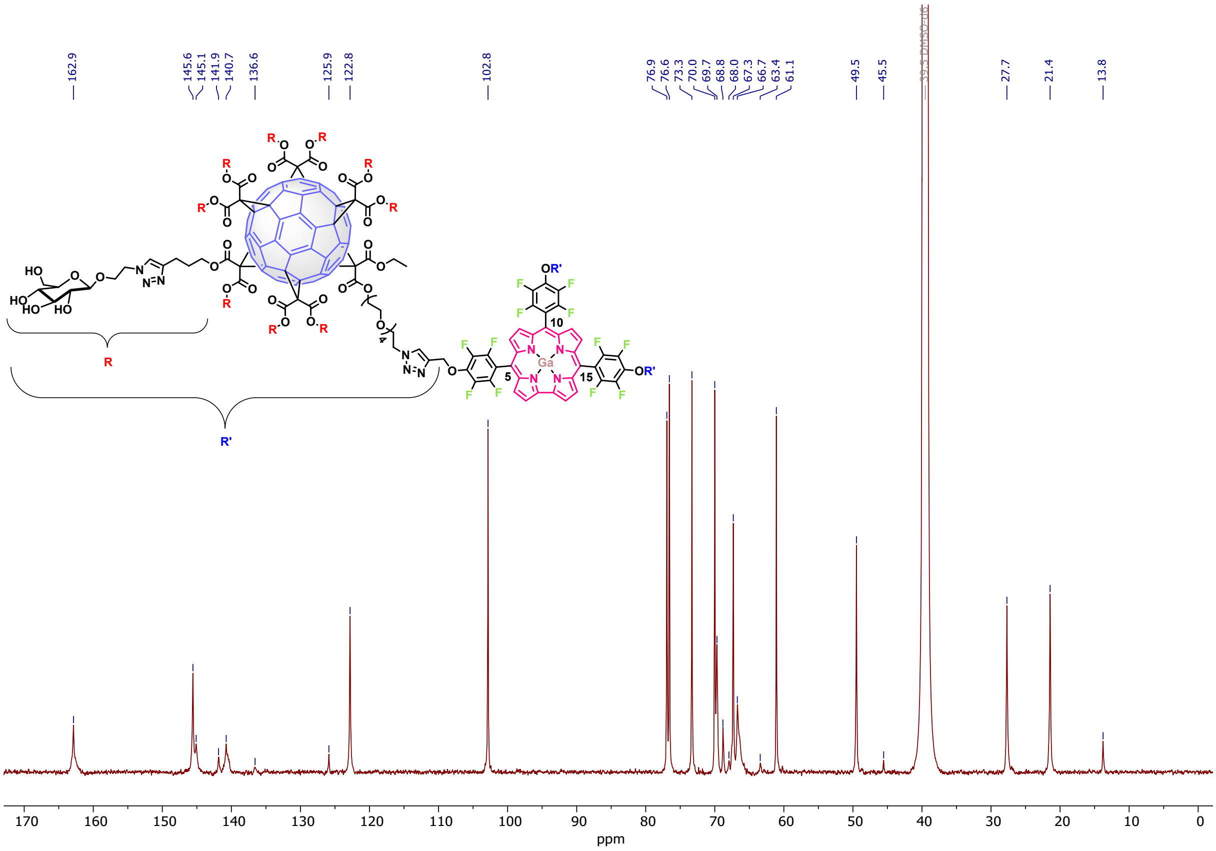

Figure S27. *^{13}C NMR spectrum of compound **13** in DMSO-d_6.*

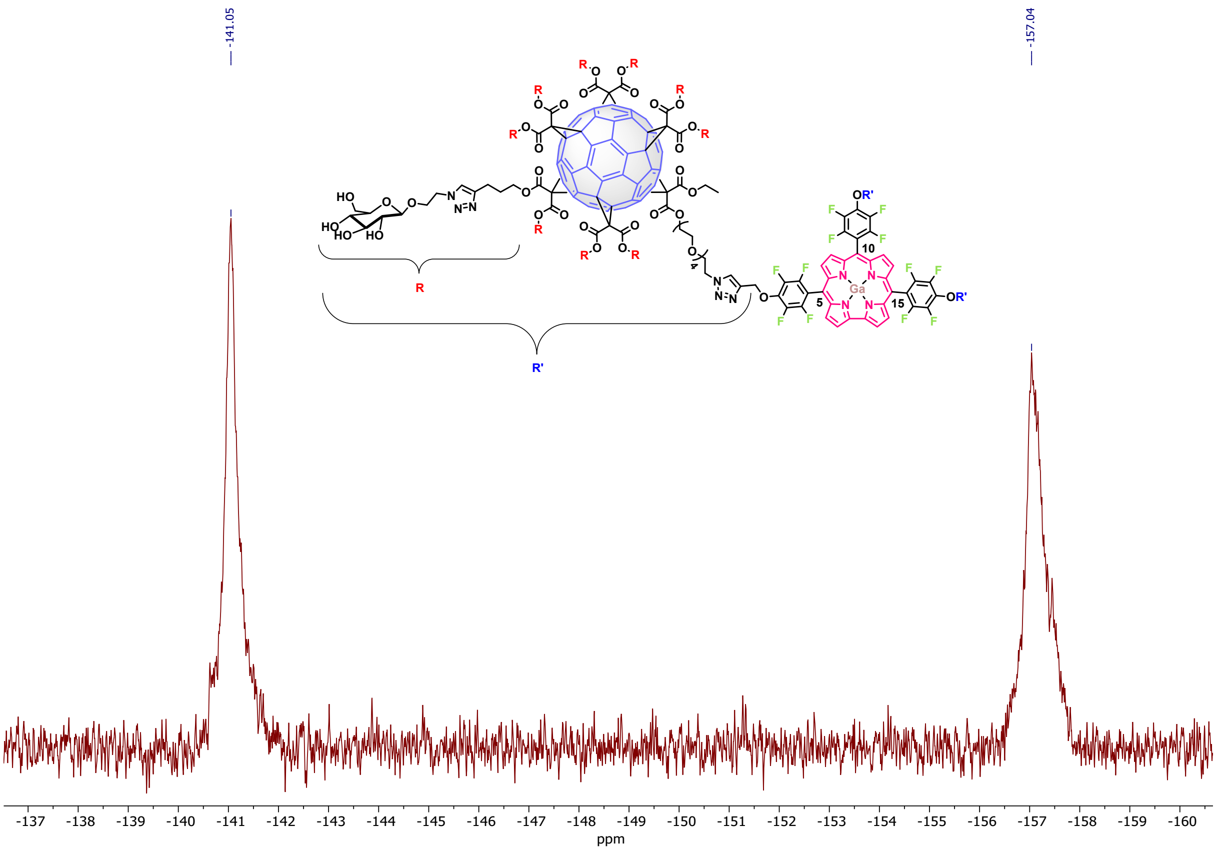

Figure S28. *^{19}F NMR spectrum of compound* ***13*** *in DMSO-d₆.*

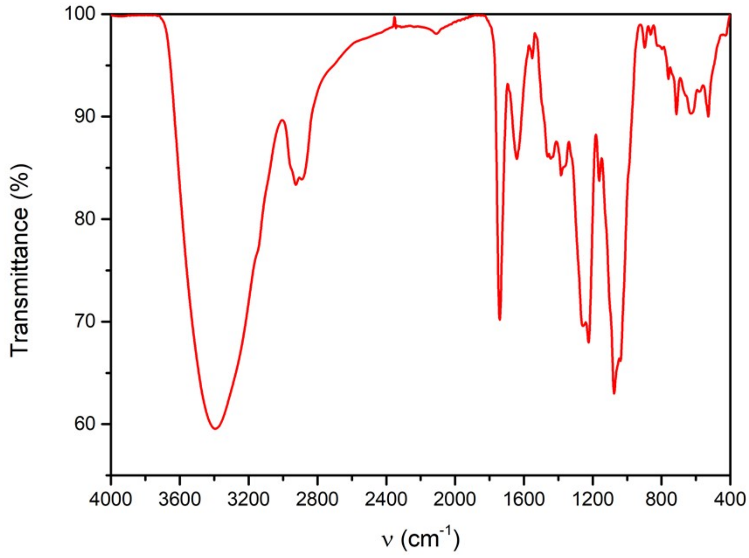

Figure S29. *IR (KBr) spectrum of compound* ***13***.

1.2. Diffusion-Ordered NMR Spectroscopy (DOSY)

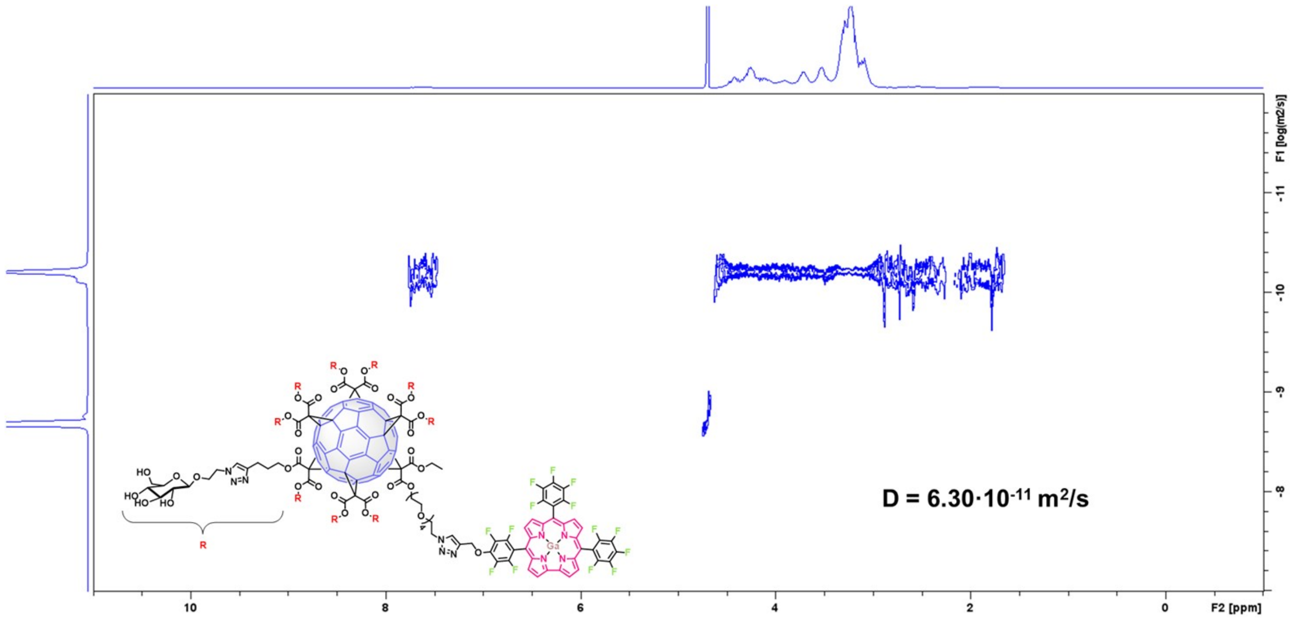

Figure S30. *DOSY-NMR spectrum of compound **12** in D_2O.*

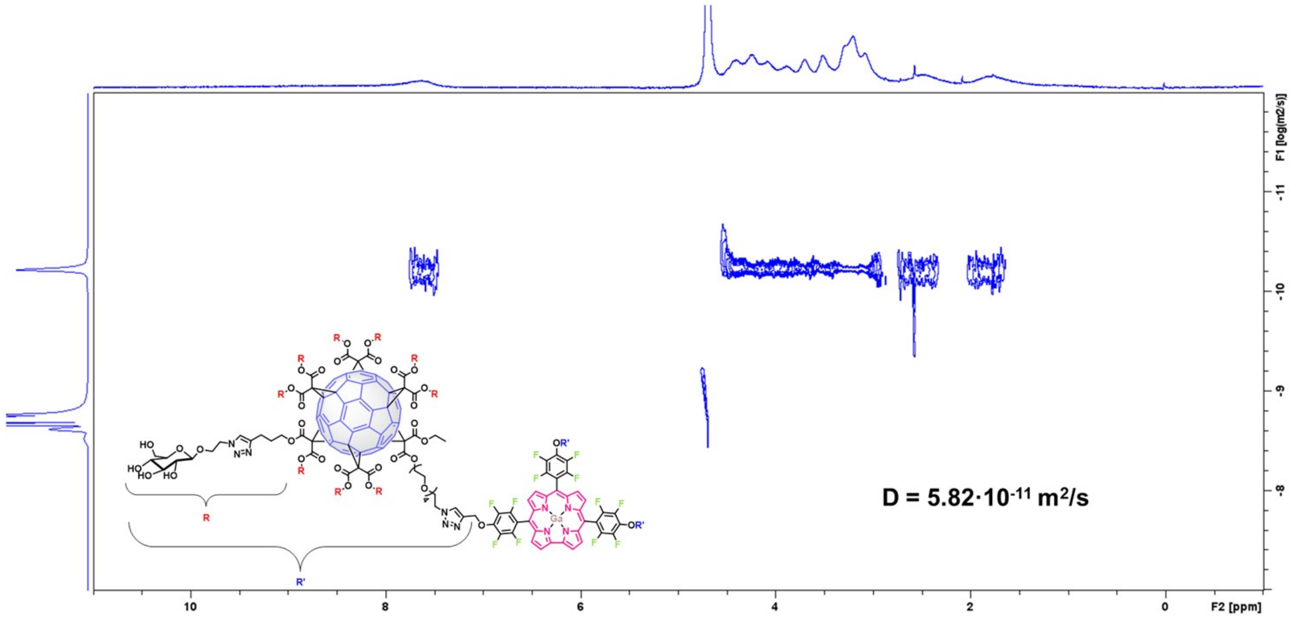

Figure S31. *DOSY-NMR spectrum of compound **13** in D_2O.*

1.3. Dynamic Light Scattering (DLS) analysis

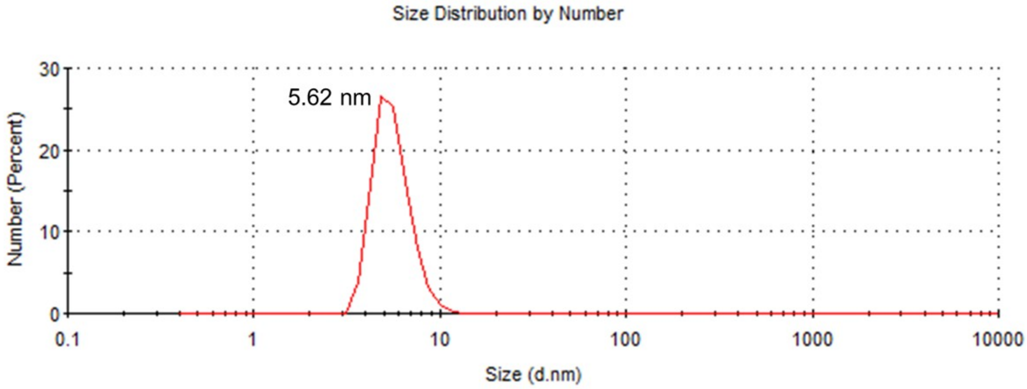

Figure S32. *DLS of compound **12** in H_2O ($1\cdot10^{-5}$ M). Intensity vs. particle number.*

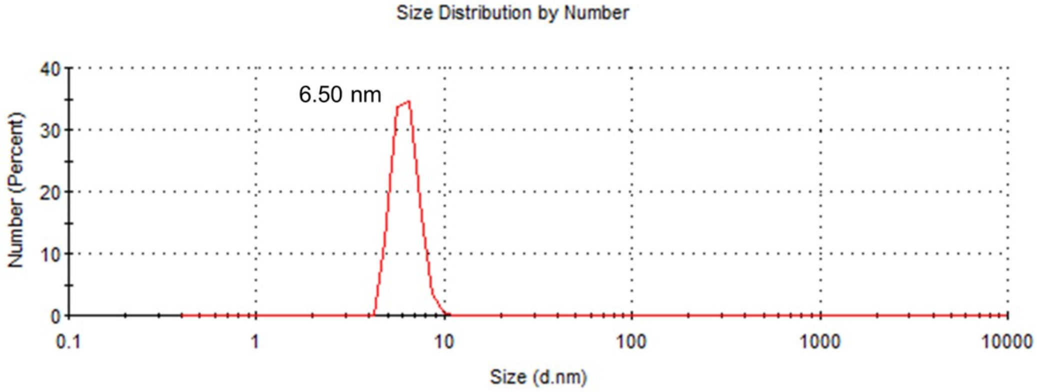

Figure S33. *DLS of compound **13** in H_2O ($1\cdot10^{-5}$ M). Intensity vs. particle number.*

1.4 Spectroscopic and photophysical characterization

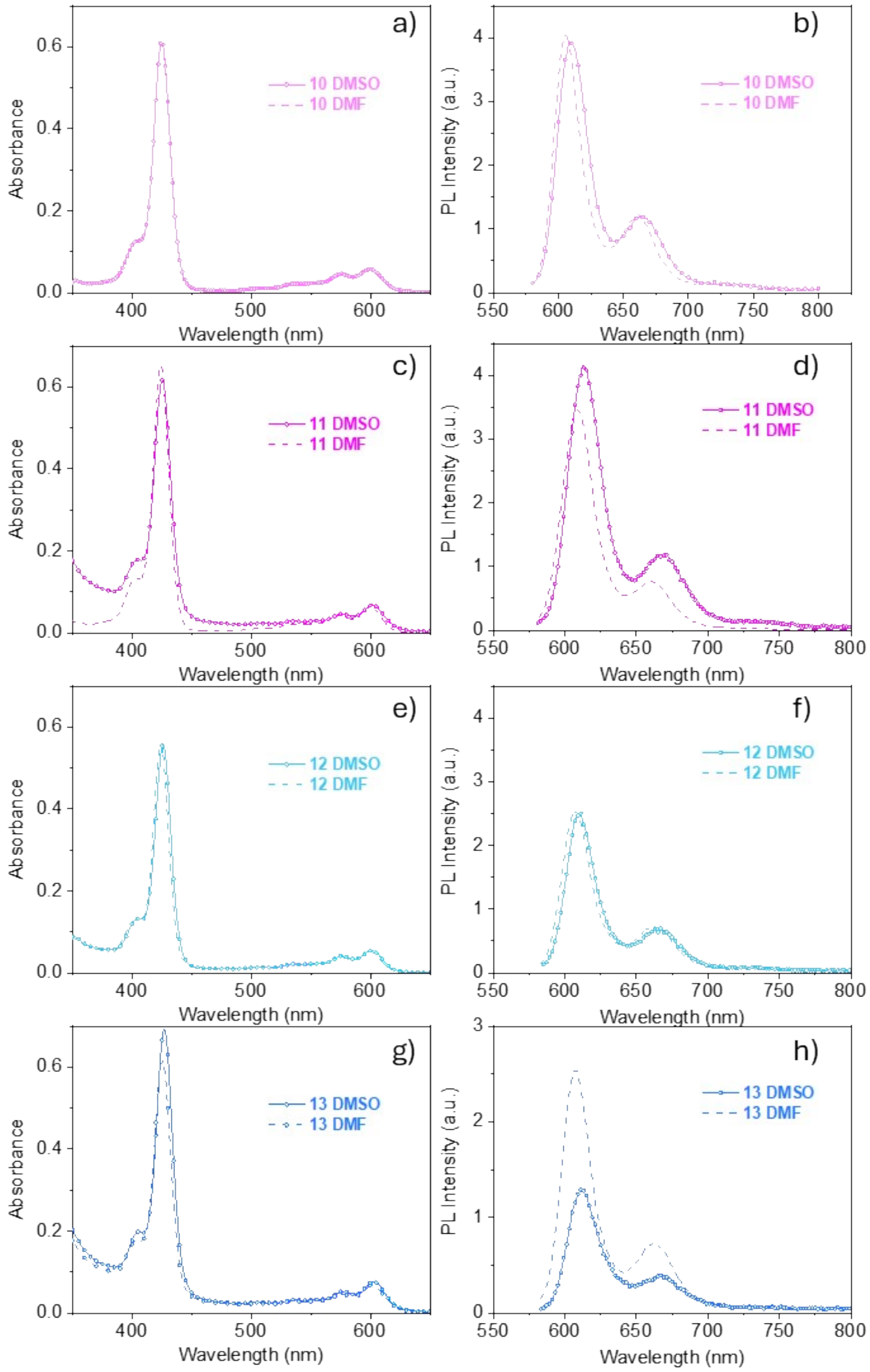

Figure S34. *UV-Vis absorption and emission spectra of the corroles (**10** and **11**) and glycofullerene-corrole conjugates (**12** and **13**) in DMSO and DMF (5 x 10^{-6} M): a,) c), e) and g) absorption spectra and b), d), f) and h) fluorescence emission spectra excited at 575 nm.*

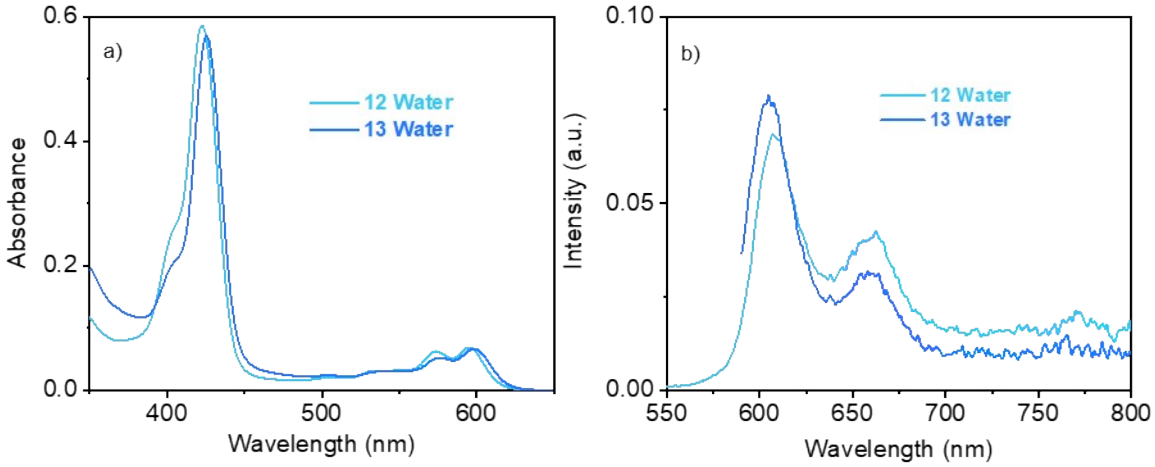

Figure S35. *UV-Vis absorption and emission spectra of the glycofullerene-corrole conjugates (**12** and **13**) in water (5 x 10^{-5} M): a) absorption spectra and b) fluorescence emission spectra:* **12** λ_{exc}*=425 nm;* **13** λ_{exc} =575 nm.

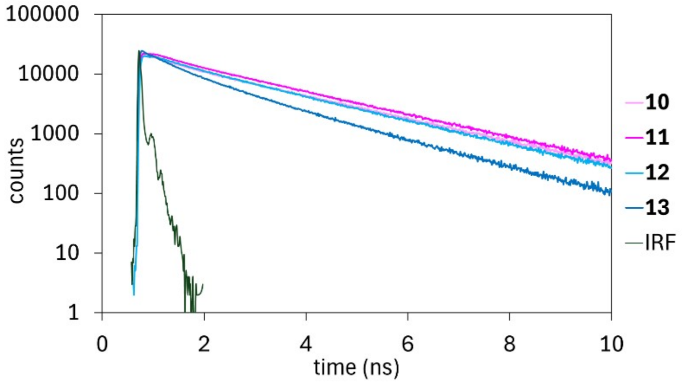

Figure S36. *Fluorescence emission decays measured at 650 nm upon excitation at 570 nm in DMF of the corroles **10** and **11** and glycofullerene-corrole conjugates **12** and **13**. The instrumental response function (IRF) is included.*

2. Biological Assays

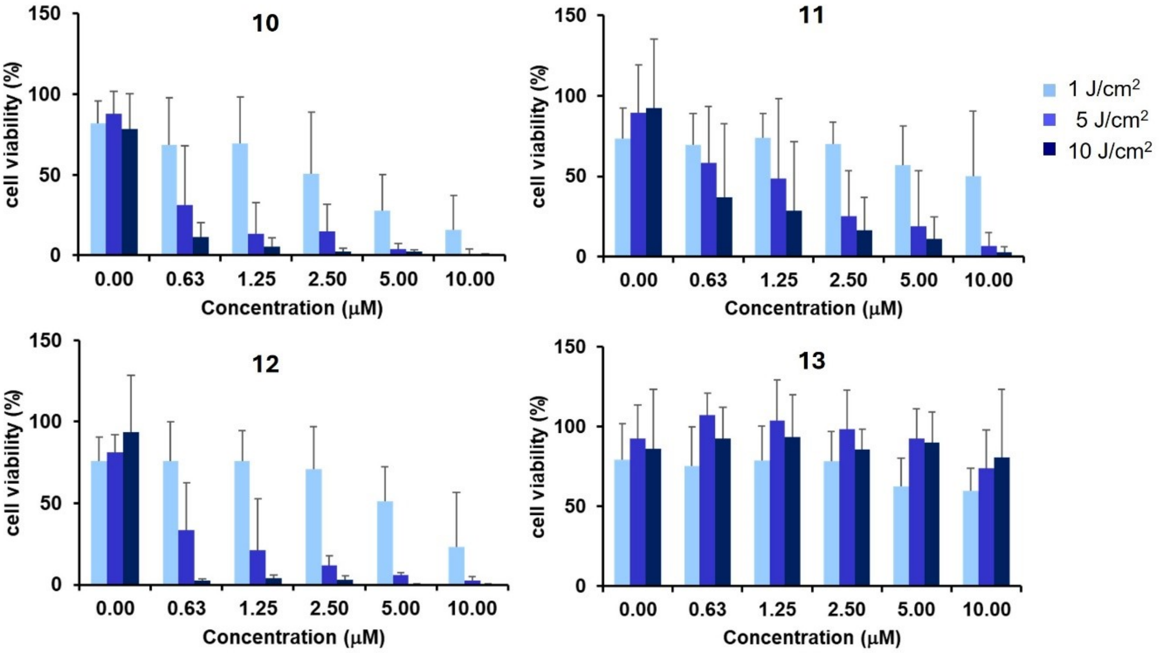

Figure S37. *Cellular metabolic viability of HeLa cells after treatment with compounds **10-13** and exposure to increasing light doses. HeLa cells were pre-incubated for 12 h with compounds **10-13** and then irradiated at a total light dose of 1, 5, and 10 J/cm². Cellular viability was assessed 24 h post-irradiation using a standard metabolic assay. Results are presented as mean ± SD normalized to dark control.*

Table S1. *IC_{50} (μM) values determined for compounds **10–13** using GraphPad Prism software. Data were analyzed by nonlinear regression with the log(inhibitor) vs. response – variable slope (four parameters) model.*

Compound	10	11	12	13
**Non-irradiated**	4.96	n.d	n.d	n.d
**1 J/cm²**	4.16	4.94	4.42	n.d

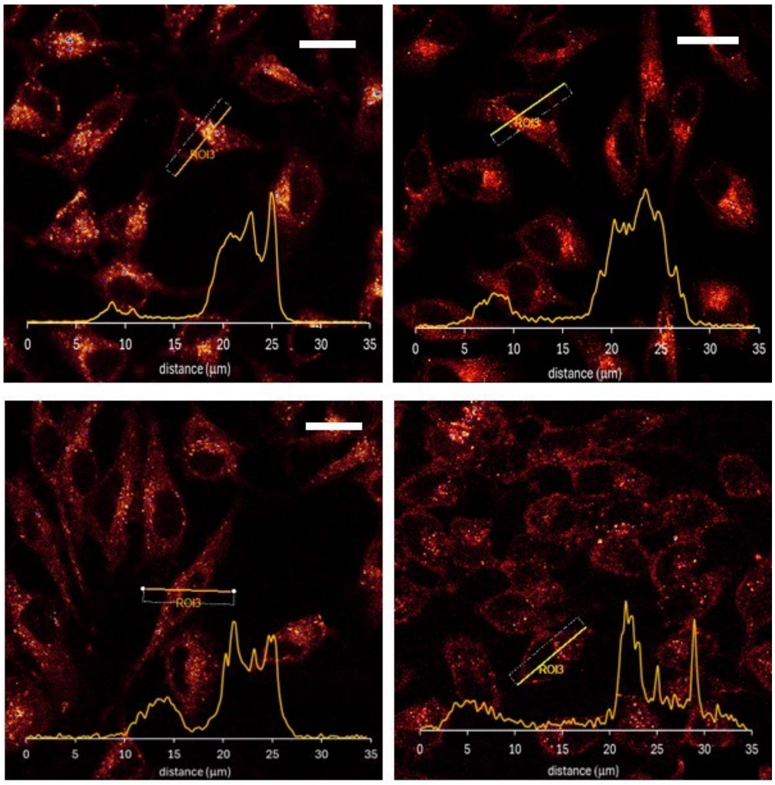

Figure S38. *Fluorescence image of the HeLa cells incubated overnight with 10 µM of each compound (**10** (top left), **11** (bottom left), **12** (top right), **13** (bottom right)) recorded upon excitation at 514 nm, with emission collected in the 600-700 nm range. The plots show the intensity profile along a region of interest crossing the nucleus of a single cell as depicted in the image. The scale bar is 20 µm.*
